# Comparing raw score difference, multilevel modeling, and structural equation modeling methods for estimating discrepancy in dyads

**DOI:** 10.3389/fpsyg.2025.1499076

**Published:** 2025-06-19

**Authors:** Amber McEnturff, Qi Chen, Robin K. Henson, Ryan Glaman, Wen Luo

**Affiliations:** ^1^Alexandria City Public Schools, Alexandria, VA, United States; ^2^Department of Educational Psychology, University of North Texas, Denton, TX, United States; ^3^Department of Educational Leadership and Technology, Tarleton State University, Stephenville, TX, United States; ^4^Department of Educational Psychology, Texas A&M University, College Station, TX, United States

**Keywords:** dyadic analysis, dyadic discrepancy, multilevel modeling, structural equation modeling, Monte Carlo simulation

## Abstract

**Introduction:**

Dyadic data analysis is commonly used in psychological research involving pairs of individuals in a nested relationship, such as parent and child, student and teacher, and pairs of spouses. There are several methods for calculating dyadic discrepancy (i.e., difference) scores, and purpose of the present study was to explore which of these methods produced the most accurate discrepancy estimates and most accurate outcome prediction.

**Methods:**

Using a Monte Carlo simulation, the present study compared three methods for estimating discrepancy scores in dyad pairs: raw score difference (RSD), empirical Bayes estimates from multilevel modeling (MLM), and factor scores from structural equation modeling (SEM). Design factors for this simulation included intraclass correlation (ICC), cluster number, reliability estimates, effect size of discrepancy, and effect size variance.

**Results:**

Results suggest discrepancy estimates from MLM had poor reliability compared to RSD and SEM methods. These findings were driven primarily by having a high ICC, high effect size variance, and low number of clusters. None of the design factors had an appreciable impact on either the RSD or SEM estimates.

**Discussion:**

RSD and SEM methods performed similarly, and are recommended for practical use in estimating discrepancy values. MLM is not recommended as it featured comparatively poor reliability.

## 1 Introduction

This study’s purpose was to determine the best way to measure the difference between members of a dyad, or two people, on a psychological construct (i.e., relationship satisfaction). Because there are several methods used in the literature and no clear consensus on the best approach, a Monte Carlo simulation compared three of these methods across a variety of potential research conditions, including variations on intraclass correlation (ICC), cluster number, reliability, effect size, and effect size variance.

Sayer and Klute (2005) defined a dyad as “two individuals” “nested in a relationship” (p. 291). Common examples of dyads include parent and child, employee and supervisor, or student and professor. Dyadic discrepancy is defined as the degree to which two individuals nested in a relationship differ on some construct of interest. The construct could be virtually any psychological or educational measure, such as depression, intelligence, or personality. For example, if each member of a heterosexual married couple completes an assessment of marital satisfaction, the scores may reveal that a wife has a much higher level of marital satisfaction than her husband. The difference in levels of marital satisfaction between the husband and wife is the dyadic discrepancy. The discrepancy score represents both the magnitude (size) and direction (which dyad member has a higher score) of the difference.

In addition to understanding the discrepancy itself, it may be useful to understand the relationship between the discrepancy and some other variable. Following the marital satisfaction scenario, the amount of discrepancy may be related to the effectiveness of couples’ counseling. Therefore, the focus of this study includes both the estimation of discrepancy and its relationship with another variable.

The importance of accurate discrepancy estimation lies in both the prevalence of research using dyads and the implications of inaccurate discrepancy estimation. Dyadic discrepancy is studied in multiple areas of psychology. For example, the acculturation gap (Costigan and Dokis, 2006) is a type of dyadic discrepancy that may occur between parent and child when one member of the dyad acculturates to a new culture at a different level than the other member. The discrepancy represents the size of the acculturation gap and the direction (whether parent or child has become more acculturated than the other). Acculturation gap may predict child maladjustment (Kim et al., 2013). In this example, accurate discrepancy estimation is important for understanding the relationship between the acculturation gap and outcomes like child maladjustment, which may then impact family interventions.

As another example, dyadic discrepancy has also been applied in end-of-life care research. For example, Schmid et al. (2010) analyzed the relationship between the discrepancy in desire for medical intervention and family demographic characteristics to learn which families were most at risk for having a large discrepancy between the patients’ actual medical care wishes and how the family perceived them. Like the acculturation example, a better understanding of the relationship between the discrepancy and family characteristics can guide recommendations about interventions that prepare families for end-of-life care decisions. Other examples discrepancy research include: marital satisfaction discrepancy related to psychological adjustment to widowhood (Carr and Boerner, 2009); informant discrepancies between reporters of child psychological behavior related to the diagnosis of the child (De Los Reyes and Kazdin, 2004); and, discrepancies between parent and child educational expectations related to adolescent adjustment (Wang and Benner, 2013). Accurate and consistent discrepancy score estimation is the first step to understanding the relationship between discrepancy and other variables.

### 1.1 Conceptualization of dyad discrepancies

Importantly, there are different ways to conceptualize dyadic discrepancy, such as idiographic versus nomothetic measures of discrepancy. The idiographic approach computes discrepancy for each dyad separately and can be compared among dyads. Following the marriage example, each couple would have its own marital satisfaction discrepancy score.

In the nomothetic approach, a single discrepancy estimate is computed across all dyads. Using marriage, the discrepancy would be a single measure, that might, for example, reflect a general trend where husbands tended to be more or less satisfied in their marriages than wives. Idiographic discrepancies can be summarized using descriptive statistics such as mean or standard deviation and used in a nomothetic approach (Kenny et al., 2006a).

The goal of a dyadic analysis might be to build generalizable knowledge about marital satisfaction (nomothetic) or to better understand the marital satisfaction of individual dyads (idiographic). As described by Steele et al. (2013), analyses could combine both nomothetic and idiographic approaches. They stated, “…we need methods that allow individual trajectories to emerge while simultaneously looking to a common point of comparison” (p. 676). The methods compared in this study output a single idiographic discrepancy measure for each dyad. The discrepancy can then be used to answer idiographic or nomothetic research questions.

Another conceptual issue is the difference between distinguishable and indistinguishable dyads (Gonzalez and Griffin, 1997). Distinguishable dyads are differentiable by some trait that is of interest in the research. In marital satisfaction, a heterosexual married couple would be considered distinguishable, for example, by role (husband and wife), gender, and potentially other variables such as employment status (where one works and one does not). Indistinguishable dyads have no distinguishing factor between them, such as identical twins. Other dyads that could be distinguishable, like husband and wife, may be treated as indistinguishable if role, gender, or other distinguishing factor is not of interest to the research study. For distinguishable dyads, both the size (how different are the members of a dyad) and direction (which dyad member has the higher score) of the discrepancy must be used in the analysis. For indistinguishable dyads, only the size of the discrepancy matters, and some statistics would be inappropriate for such data. For example, Pearson *r* should not be computed for indistinguishable dyads because *r* requires paired data with specific groups, where each score in the pair must belong to a particular group. Pearson *r* could mathematically be computed as a measure of association between a group of dyads, but *r* would depend on how each dyad member was assigned to a group, which would be an arbitrary decision that alters the value of *r* when changed. Therefore, it is important to ensure measures computed for dyadic data appropriately take distinguishability into account.

Another issue impacting the computation of dyadic discrepancy is whether a single score or composite scale score is used. In some discrepancy models, the construct of interest is represented by a single score from each dyad member, which may be a single item score or a composite scale score generated from multiple items in a separate analysis of the measurement model. Conversely, some models, such as structural equation models, use item-level data and incorporate the measurement model and discrepancy model into one analysis (Newsom, 2002). The scope of this research is limited to the single-score case. Whether a single score or item-level data is used, measurement invariance is assumed before calculating discrepancy scores. Confirmation of measurement invariance ensures that measures are indeed tapping the same construct when used with two different populations (Russell et al., 2016).

In summary, when choosing discrepancy calculation methods, it is important to consider whether the research is idiographic or nomothetic in nature, whether dyads are distinguishable or indistinguishable within the context of the research question, and what kind of numerical data will be used to compute the discrepancy (a single score or multiple scores).

### 1.2 Discrepancy estimation methods

Three methods for estimating discrepancy were identified that fit into the theoretical framework described above; that is, they provide an idiographic measure of discrepancy (an individual score for each dyad), they may be used for distinguishable or indistinguishable dyads, and they use the composite score rather than individual item scores. The three methods are the raw score difference (RSD), the empirical Bayes discrepancy (EBD) estimate from multilevel modeling (MLM), and the factor score from structural equation modeling (SEM). In this section, each method is described in detail, and reasons for not including other discrepancy methods are also provided. Throughout this section, the symbols *X* and *Y* are used to represent scores from dyad members *A* and *B*, respectively.

#### 1.2.1 Raw score difference

The RSD is computed by subtracting one raw score from another, as shown in Equation 1:


(1) RSD=X−Y

The RSD is easily interpretable; a value of 0 represents lack of discrepancy between the dyad members (Guion et al., 2009).

RSD has been criticized for having low reliability (Cronbach and Furby, 1970). Reliability is the overall consistency of a measure and, according to classical test theory, is the true score variance divided by observed score variance (DeVellis, 2006). In applied research, where it is not possible to know true score variance, reliability is estimated using methods such as test–retest or inter-rater reliability. Using Monte Carlo simulation methods, however, it is possible to compute the true measure of reliability because both true score and observed score variances are known.

The formula for estimating reliability of RSD based on its components (raw scores from each dyad member) has several variations (Lord, 1963). The formula suggested by Lord assumes uncorrelated error variance, which is likely to be violated in dyads due to the dependency of dyad members on one another. Thus, to illustrate the reliability issue with RSD scores, a formula allowing correlated error variances was used. Williams and Zimmerman’s (1977) formula was used in a pre-test post-test design context, but it can be interpreted in the dyadic discrepancy context by thinking of *X* and *Y* as scores from each member of a dyad, respectively. The formula for reliability is shown in Equation 2:


(2) $$\rho_{\mathrm{DD}\prime }=\frac{\begin{array}{l} \rho_{\mathrm{XX}\prime }\text{VarX}+\rho_{\mathrm{YY}\prime }\text{VarY}-2\rho_{\mathrm{XY}}\sigma_{X}\sigma_{Y}\\ +2\rho (E_{X},E_{Y})\sigma_{X}\sigma_{Y}\sqrt{\left(1-\rho_{\mathrm{XX}\prime })(1-\rho_{\mathrm{YY}\prime }\right)} \end{array}}{\text{VarX}+\text{VarY}-2\rho_{\mathrm{XY}}\sigma_{X}\sigma_{Y}}$$

where *ρ_DD’_* is the reliability of RSD, *ρ_XX’_* and *ρ_YY’_* are the reliabilities of *X* and *Y* (e.g., reliabilities of the raw scores for dyad member *X* and dyad member *Y*, respectively), Var *X* and Var *Y* are the variances of *X* and *Y*, respectively, *ρ_XY_* is the correlation between *X* and *Y*, *σ_X_* and *σ_Y_* are the standard deviations of *X* and *Y*, respectively, and *ρ*(*E_X_, E_Y_*) is the correlation between the error variances of *X* and *Y*. As demonstrated in this formula, anything that makes the numerator larger in the formula above increases the reliability of RSD, thus mitigating the reliability issue of RSD and making RSD a viable option for discrepancy estimation. These include higher reliabilities of *X* and *Y* and smaller correlation between *X* and *Y*.

Another feature of this formula is that, as long as *ρ_XY_* is positive, the reliability of RSD cannot be greater than the average of the reliabilities of *X* and *Y* (Chiou and Spreng, 1996). This is the primary argument against RSD because, in some cases, reliability of RSD is actually lower than the reliabilities of *X* and *Y*, but reliability of RSD is never higher. However, Zumbo (1999) argued that because there are situations where the reliability of difference scores is not an issue, RSD should not be ruled out in every instance. Therefore, despite the potential reliability issues, the RSD was evaluated as part of this study. The RSD is expected to have lower reliability in cases where reliability of *X* and *Y* are lower or *X* and *Y* are highly correlated.

#### 1.2.2 Empirical Bayes (EB) estimate from MLM

In dyadic research, MLM can be used to estimate the average intercept and slope across all dyads as well as the within-dyad intercept and slope. In MLM, parameters are estimated using EB estimation instead of the traditional ordinary least squares (OLS) estimation method. The dyad-level slope is one of the parameters estimated in the model described below. The dyad-level slope is the idiographic discrepancy score, which is referred to as EBD in this study.

As described by Kim et al. (2013), the following MLM was used to generate the EBD:


(3a) $$\text{Level}\;1:Y_{\mathrm{ij}}=\beta_{0j}+\beta_{1j}{\left(\text{report}\right)}_{\mathrm{ij}}+\varepsilon_{\mathrm{ij}}$$

(3b) $$\text{with}\;\varepsilon_{\mathrm{ij}}∼N(0,\sigma^{2})$$

(4a) $$\text{Level}\;2:\beta_{0j}=\gamma_{00}+u_{0j}$$

(4b) $$\beta_{1j}=\gamma_{10}+u_{1j}$$

(4c) $$\text{with}\;[u_{0j}u_{1j}]∼\mathrm{MVN}([0,0],[\tau_{00}\tau_{01}\tau_{10}\tau_{11}])$$

(5) $$\text{Combined model}:Y_{\mathrm{ij}}=\gamma_{00}+{\gamma_{10}}^{*}\text{repor}t_{\mathrm{ij}}+(u_{0j}+{u_{1j}}^{*}\text{repor}t_{\mathrm{ij}}+\varepsilon_{\mathrm{ij}})$$

*Y_ij_* represents the score for each individual *i* within the same dyad *j.* “Report” is a dichotomous indicator with a value of −0.5 if the score is reported by dyad member *A*, and 0.5 if reported by dyad member *B*. *β_0j_* is the mean score between *X* and *Y* for each dyad *j*, and *β_1j_* is the discrepancy score between *X* and *Y* for each dyad *j*. *ε_ij_* is the unique effect associated with individual *i* nested within dyad *j* (i.e., measurement error). *γ*_00_ is the mean score across all dyads, and *γ*_10_ is the mean discrepancy score across all dyads. *u_0j_* is the unique effect of dyad *j* on the mean score, and *u_1j_* is the unique effect of dyad *j* on the mean discrepancy score.

This random-coefficient model was fitted using the EB estimation procedure (Raudenbush and Bryk, 2002). The EB estimates of *β*_1j_ (i.e., discrepancy in each dyad, or EBD) of the model were saved.

The model requires input of measurement error for the observed scores from each dyad member in order to sufficiently identify the model (Cano et al., 2005). The formula used to calculate measurement error (i.e., *r_ij_*) is given in Equation 6:


(6) $$\mathrm{ME}=(1-\alpha )*\sigma^{2}$$

where *α* is the reliability of the measure, and *σ^2^* is the variance of all scores within dyads.

The EBD may be a better estimate of discrepancy. EB estimates are also known as “shrinkage” estimates (Raudenbush and Bryk, 2002). The equation for the EB estimate is:


(7) $${\hat{\beta }}_{1j}^{\mathrm{EB}}=\lambda_{j}{\hat{\beta }}_{1j}^{\mathrm{OLS}}+(1-\lambda_{j})\gamma_{10}$$

where *λ_j_* is the reliability of the OLS estimate, and *γ*_10_ is the overall slope across all dyads. The EB estimate is shrunken based on the reliability of the OLS estimate (*λ_j_*) which is defined in Equation 8:


(8) $$\lambda_{j}=\frac{\mathrm{Var}({\hat{\beta }}_{1j}^{\mathrm{OLS}})}{\mathrm{Var}({\hat{\beta }}_{1j}^{\mathrm{OLS}})+\sigma^{2}/n_{j}}$$

where $\mathrm{Var}({\hat{\beta }}_{1j}^{\mathrm{OLS}})$ represents the variance of the OLS estimates and *n_j_* = 2 in a dyad. Reliability increases as the variance of the OLS estimates increases, the level-1 residual variance decreases, or *n_j_* increases.

The OLS estimate is weighted by reliability, and therefore counts less toward the EB estimate as reliability decreases. Meanwhile, the overall slope (*γ*_10_) is weighted by one minus the reliability, such that as reliability decreases, the overall slope is weighted more. Including both the OLS estimate and the overall slope, adjusted for reliability, results in an optimal weighted combination of the two (Raudenbush and Bryk, 2002). Another way of viewing the EB estimate is as a “composite of the sample slope estimate (*γ*_10_) and the predicted value of individual’s slope estimate $\left({\hat{\beta }}_{1j}^{\mathrm{OLS}}\right)$ ” (Stage, 2001, p. 92).

When reliability estimates are low, as is the case with small cluster sizes such as dyads, the EB “borrow strength from all of the information…in the entire dataset to improve the estimates for dyad discrepancy scores” (Kim et al., 2013, p. 905). In addition, variance in the scores is divided into two parts: (a) variance associated with dyads; and (b) variance associated with individual members in the dyads (i.e., measurement error variance) (Kim et al., 2013). The discrepancy estimates have measurement error partialed out and may be a more accurate estimate of the discrepancy.

On the other hand, a drawback of EB estimates is that they may “over-shrink” the estimates of random coefficients when cluster size is very small and lead to under-estimates of the posterior variance of the random coefficients (Raudenbush, 2008). As a result, when EB estimates are used as predictors in a regression analysis, their raw regression coefficients and standard error estimates might be inaccurate, especially when the variance of EB estimates differ significantly from that of the true discrepancies.

#### 1.2.3 SEM discrepancy

**Figure 1:**
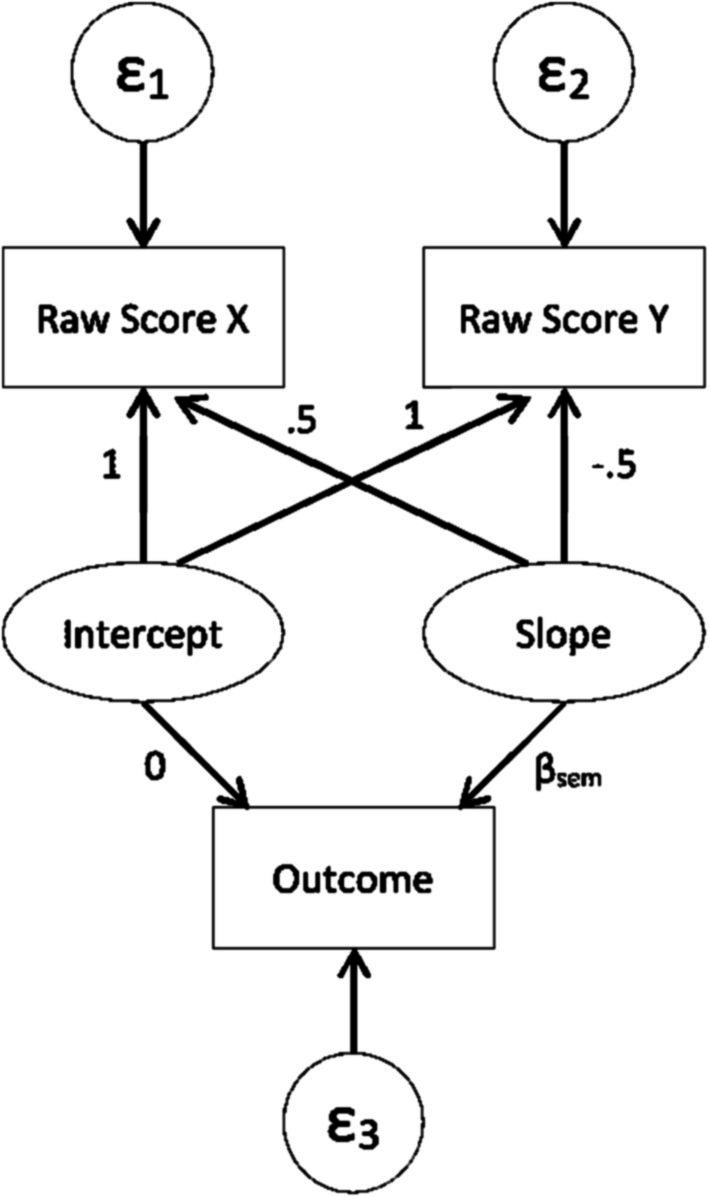
SEM path diagram for predicting an outcome with discrepancy score based on Newsom (2002).

SEM can be used to estimate discrepancy by fitting the model shown in Figure 1, which is based on Newsom (2002). In the SEM discrepancy model, the latent intercept and slope predict the individual scores of each dyad member. Paths from the slope to individual scores are fixed to 0.5 and −0.5, so that intercept reflects the average score of both dyad members. The paths from intercept to individual scores are fixed to 1. The SEM dyadic discrepancy is indicated by the latent slope in Figure 1. Fitting this model using SEM would typically result in a single estimate for the latent slope that represents all dyads. However, it is possible to specify options in some software packages (such as PROC SCORE in SAS, or SAVEDATA in Mplus) that would save idiographic estimates of the latent slope. These individual estimates of the latent slope serve as the discrepancy estimates. One primary benefit of using SEM is the model’s flexibility, such as the ability to incorporate correlated measurement errors into the model (Newsom, 2002).

#### 1.2.4 Other discrepancy estimation methods not included in the present study

Two other methods found in discrepancy research were excluded from this study. One was the standardized score difference, notated as difference in z (DIZ). To calculate DIZ, each dyad member’s score is converted to a z score, and one member’s score is subtracted from the other (De Los Reyes and Kazdin, 2004). However, the standardization of scores prior to computing a discrepancy changes the interpretation of the discrepancy (Guion et al., 2009). Compared with the RSD method, a DIZ score of 0 does not mean perfect agreement between dyads, but rather that both dyad members have average scores within their respective distributions.

The second method excluded from this study was the OLS residual method (abbreviated as “RES” to indicate residual). RES involves using one member’s rating to predict the other’s in a linear regression, and outputting the residual for each dyad to serve as a discrepancy (De Los Reyes and Kazdin, 2004). This score is typically standardized into a z score before use. The RES score is affected by the correlation between dyad members’ scores (De Los Reyes and Kazdin, 2004). Say for example dyad member Y’s score is predicted by dyad member X’s score in a linear regression, and the standardized residual (RES) is output. The correlation between the independent variable X and RES is always 0. Larger correlations between dyad members X and Y mean weaker relationships between the Y and RES, while smaller correlations between X and Y mean stronger relationships between the Y and RES. This is true because, the more variance in Y explained by X, the less variance there is left unexplained (i.e., the variance of residual), and the less Y is related to that residual. When there is not much variance in Y explained by X, there is a lot of Y-related variance leftover.

### 1.3 Possible factors influencing discrepancy estimation

As discussed above, this study includes three methods of estimating dyadic discrepancy. In addition there are several data characteristics (i.e., design factors) which may impact the estimation of dyadic discrepancy. Based on review of applied and methodological research, these include ICC, cluster number (number of dyads), reliability of measurement, and effect size and effect size variance of the discrepancy. Each of these is discussed in more detail in this section.

#### 1.3.1 Intraclass correlation

Nonindependence is a key consideration in dyadic data. Nonindependence means that the scores from two dyad members may share similarity more than scores from people not within the same dyad. Thus, the scores violate the assumption of independence of observations under the general linear model framework. The degree of nonindependence may impact the estimation of dyadic discrepancy. Ignoring the nonindependence of observations and analyzing data as though they are independent has implications for the accuracy of standard error estimates (Chen et al., 2010). The standard errors for predictors at the ignored level may be underestimated, inflating the Type I error rate. Conversely, the standard error of a predictor below the ignored level may be overestimated, reducing the statistical power of the analysis (Luo and Kwok, 2009; Moerbeek, 2004).

There are several methods for measuring nonindependence. The unconditional ICC can be used to measure nonindependence. The unconditional ICC is computed as shown in Equation 9 (Raudenbush and Bryk, 2002):


(9) $$\rho =\frac{\tau_{00}}{\sigma^{2}+\tau_{00}}$$

where *τ*_00_ is the variance between dyads (i.e., how much the average of the two scores within the dyad varies among dyads) and *σ^2^* is the variance within dyads. ICC is interpreted as the proportion of variance in the individual scores that is between dyads. In other words, it is how much of the variance in level-1 scores is explained at the dyad level. Higher values of ICC indicate a stronger clustering effect, or a higher level of nonindependence. In addition to *unconditional ICC*, there is also the *conditional ICC*, which is computed after predictors are included in the HLM model (Raudenbush and Bryk, 2002).

The level of nonindependence has shown varying impacts in studies. One study revealed no effect of ICC on parameter estimates in a multilevel model with one predictor at level one and one predictor at level two (Maas and Hox, 2005). Low ICC may help overcome small cluster numbers, resulting in more accurate parameter and standard error estimates (Maas and Hox, 2005). Another study revealed underestimated standard errors when ICC was higher (Krull and MacKinnon, 2001). Low ICC is expected to result in more accurate discrepancy estimation in the current study according to the reliability equation from Williams and Zimmerman (1977).

#### 1.3.2 Cluster number

Cluster number, or the number of dyads, varies widely in applied research and has been shown to impact the accuracy of parameter estimation (Maas and Hox, 2005) and the power for detecting statistical significance (Chen et al., 2010). While fixed effects are consistently accurate despite cluster number, standard error estimates are typically biased when cluster number is less than 100 (Clarke and Wheaton, 2007; Maas and Hox, 2005). Standard error estimates typically improve as cluster number increases (Maas and Hox, 2005).

#### 1.3.3 Reliability

Reliability, as a design factor, is operationalized in this study as variance of scores among a group of dyad members in a sample, i.e., the variance of all *X* raw scores from dyad member A, compared with the variance of true scores. Increased reliability is expected to result in more accurate parameter estimates.

#### 1.3.4 Effect size and effect size variance of discrepancy

The size of the discrepancy between dyad members, and the variation of effect size among dyads, may also impact its estimation. In a previous study about dyadic discrepancy estimation, accuracy of EBD estimates was better when effect size was lower (McEnturff et al., 2013). Furthermore, when using discrepancy as an independent variable in regression, the accuracy of intercept and slope estimates was better when effect size was higher.

### 1.4 Gaps in current literature

Historically, several studies have examined various approaches and design factors for discrepancy estimation. De Los Reyes and Kazdin (2004) compared RSD to two other methods, the standardized score and residual score methods, using one empirical dataset. Although De Los Reyes and Kazdin thought for the purposes of their research that the standardized score method was most appropriate, in their conclusion, they stated, “However, there may be other instances in which other measures may be conceptually and methodologically optimal” (p. 334). They also noted that, “Our goal was to convey that an accumulating body of research cannot be expected to produce consistent results because the measures used among the studies are not interchangeable” (p. 334). A thorough analysis of existing discrepancy methods would aid in comparability of results produced in discrepancy research. Furthermore, De Los Reyes and Kazdin used empirical data. As explained later, a statistical simulation has some advantages when comparing various methods of estimation.

Kenny et al. (2006a, 2006b) have published substantial amounts of literature about dyadic data. However, they focus on nomothetic approaches. The idiographic measurement of dyadic discrepancy still needs investigation, as noted in their book. It is important to study approaches that work for both idiographic and nomothetic research. Nomothetic research may be more useful in moving theory forward, but idiographic scores have immediate clinical use for understanding how a particular dyad fits within a theory and using that knowledge to guide interventions for the dyad.

More recent research has also illustrated gaps and highlighted the need to further methodologically examine various approaches to discrepancy estimation in dyadic analysis. Although the RSD approach has been historically criticized for its low reliability (Cronbach and Furby, 1970), recent researchers have continued to debate its use, both highlighting its problems (Laird, 2020) and defending its use in certain contexts (Campione-Barr et al., 2020). Furthermore, although the EBD estimation approach using MLM has seen recent use in empirical literature (Bar-Sella et al., 2023), there has been little-to-no recent methodological research examining this approach. Lastly, although SEM-based discrepancy estimation methods see continued use in both empirical (Barooj-Kiakalaee et al., 2022) and methodological (Sakaluk et al., 2025) literature, little simulation work has been conducted recently comparing this estimation approach with other competing approaches. Overall, these various gaps demonstrate the need to examine and compare these types of approaches to discrepancy estimation in dyadic analysis.

Finally, the importance of discrepancy estimation stems from the high-stakes topics studied using dyadic data, including topics related to family functioning, adolescent adjustment, and end-of-life-care. Dyads are the building blocks of interpersonal relationships, and better understanding of dyads can lead to stronger theories to support the well-being of individuals and families.

### 1.5 Purpose of the current study

Given the potential implications of inaccurate discrepancy estimation, and the lack of research comparing the methods, the following questions are addressed with this study. First, of RSD, EBD, and SEM, which method generates the most accurate estimate of discrepancy? Second, of the three methods, which allows the most accurate prediction of an outcome? Finally, what is the impact of the design factors ICC, cluster number, reliability, effect size, and effect size variance on the accuracy of estimates and prediction?

## 2 Method

A Monte Carlo simulation study was conducted, in which the true scores are generated first, then error added in order to create observed scores. Once the analysis was conducted on the observed scores, the results could be compared to the true scores to assess the performance of the statistic. This will further the work of De Los Reyes and Kazdin, whose empirical study did not allow the comparison to the true score.

### 2.1 Design factors

To enhance the external validity of the simulation study results, findings from a past literature review (McEnturff et al., 2013) helped set the levels of design factors that are found in real life research involving dyads. In their review, McEnturff et al. evaluated the following literature: Baumann et al. (2010), Blood et al. (2013), Costigan and Dokis (2006), Crouter et al. (2006), Gulliford et al. (1999), Kim et al. (2009), Lau et al. (2005), Leidy et al. (2009), McHale et al. (2005), Stander et al. (2001), and Wheeler et al. (2010). This review led to the following design factors.

#### 2.1.1 Conditional ICC

To avoid conflation of the unconditional ICC and the standardized average discrepancy within dyads, we manipulated the conditional ICC when “report” is included in the data generation model depicted in Equations 3a–5. The conditional ICC is independent of the discrepancy within dyads (*β*_1*j*_). From here onward, ICC all refers to “conditional ICC.”

Data reflected conditional ICC values of 0.1, 0.3, and 0.5 with “report” as the only predictor. Studies using MLM in complex survey designs have typical ICC values ranging from 0 to 0.3 (Gulliford et al., 1999). Research involving dyads has shown ICC values as high as 0.49 (Blood et al., 2013).

#### 2.1.2 Cluster number

Data sets with cluster numbers of 50, 150, 250, and 400 were generated. Cluster numbers found in the review ranged from 68 (Stander et al., 2001) to 399 (Kim et al., 2009). Cluster numbers were distributed throughout that range.

#### 2.1.3 Reliability

For distinguishable dyads, data were generated with both matching, set at 0.7 and 0.8, and mismatched reliabilities of 0.7 for one dyad member and 0.8 for the other. Commonly found levels of reliability in the literature review were similar to the minimum values accepted as adequate in education, ranging from coefficient alpha of 0.67 (Wheeler et al., 2010) to 0.96 (Baumann et al., 2010).

#### 2.1.4 Effect size of discrepancy

For this study, Cohen’s *d* was 0.2, 0.5, and 0.8 to reflect widely used benchmarks for small, medium, and large effects (Cohen, 1988). It should be noted that these benchmarks are simply a rule of thumb and should not be the sole factor for evaluating effect size in any study. Effect sizes must be interpreted within the context of the study topic and methods. Effect sizes varied widely in the literature review, from *d* = 0.05 to *d* = 1.34.

#### 2.1.5 Effect size variance

Variance of discrepancies among dyads was set to 0.5 and 1. Literature reporting the variance of effect sizes was scant.

### 2.2 Data generation

A program was written and executed in SAS 9.4 to generate simulated data across a set of study conditions to examine bias and reliability of discrepancy estimates and their use in prediction. As described in the literature review, design factors included variations on nonindependence (ICC), cluster number, reliability, effect size, and effect size variance. The design factors are summarized in Table 1. A total of 216 simulation conditions were represented, with 1,000 replications generated for each condition, for a total of 216,000 datasets (Arend and Schäfer, 2019). After the data were generated, discrepancy estimates for all three methods (RSD, EBD, and SEM) were computed and used in subsequent evaluation analyses described below. Discrepancy estimates from the EBD and SEM methods were generated using the full-information maximum likelihood (FIML) estimation method. All analyses were conducted in SAS except the computation of SEM, which was computed using MPlus. The MPlus SAVEDATA option enabled the export of factor scores to use as the SEM discrepancy. The syntax for data generation and analysis in SAS and MPlus can be found in Appendix 1.

**Table 1:** Study conditions.

ICC			Reliability			Effect Size		Sample
ICC	Between Variance	Within Variance	Reliability	Error Variance 1	Error Variance 2	Effect Size	Effect Size Variance	*n*
0.1	0.1	0.9	0.7	0.43	0.43	0.2	0.5	50
0.3	0.3	0.7	0.8	0.25	0.25	0.5	1	150
0.5	0.5	0.5	0.7 / 0.8	0.43	0.25	0.8		250
								400

Data were generated for both indistinguishable and distinguishable dyads. However, the regression models appropriate for indistinguishable and distinguishable dyads are not the same. For indistinguishable dyads, the outcome, *Z*, was generated using arbitrary values for the slope (0.8) and intercept (0.5), as shown in the equation below:


(10) $$Z=.5+.8|X_{\text{true}}-Y_{\text{true}}|+\text{rannor}(0)$$

where *X*_true_ and *Y*_true_ are the true scores for each dyad member and the absolute value of the difference between these true scores is the discrepancy score.

However, for distinguishable dyads, the direction of the discrepancy is lost when using the absolute value of the discrepancy, such as in the indistinguishable dyad case. For example, in the parent–child dyad example, it is useful to know not only how different parents and children are, but which dyad member scores higher or lower on the construct of interest. Therefore, a different regression model must be used for distinguishable dyads.

A solution to this problem is shown in Equation 11, related to the method used by Wang and Benner (2013):


(11) $$Z=b_{0}+b_{1}|X-Y|+b_{2}W+b_{3}|X-Y|W+e$$

Here, |*X – Y*| is the absolute value of the discrepancy, W is a dichotomous indicator of the direction of the discrepancy (equal to 0 if *X < Y* and 1 if *X > = Y*), and the third predictor is the interaction between them. Following this, the outcome for distinguishable dyads in this study was generated as follows:


(12) Z=.5+.8∣X−Y∣+.5W+.2∣X−Y∣W+e

### 2.3 Evaluation of methods

The outcome of interest in this study was the bias of parameters estimated and their standard errors. The various values that were used to evaluate the discrepancy estimation methods are described in this section. Table 2 includes the equations used to compute the evaluation measures.

**Table 2:** Equations for evaluating methods for dyadic discrepancy.

Measure	Equation	Description
Absolute bias (AB) for discrepancy estimates and parameter estimates	$\mathrm{AB}=|({\hat{\theta }}_{\mathrm{est}}-\theta_{\mathrm{pop}})|$	${\hat{\theta }}_{\mathrm{est}}$ is the mean of the estimated discrepancy score across the replications and $\theta_{\mathrm{pop}}$ is the true parameter value (i.e., true discrepancy score).
Reliability of discrepancy estimates	$\frac{\sigma_{\text{true}}^{2}}{\sigma_{\mathrm{est}}^{2}}$	$\sigma_{\text{true}}^{2}$ is the variance of the true discrepancy scores, and $\sigma_{\mathrm{est}}^{2}$ is the variance of the discrepancy estimates.
AB for standard errors	$SE_{\mathrm{est}}-\text{Mean}(SD_{\mathrm{est}})$	$SE_{\mathrm{est}}$ is the standard error of the estimate, and $SD_{\mathrm{est}}$ is the standard deviation of the corresponding parameter estimates across the 1,000 iterations of each simulation condition.
Power for the slope estimate in prediction	$\frac{\text{Number of estimates with}\;p<.05}{\text{Total number of estimates}\;(1000)}$	The proportion of statistically significant estimates out of the total number of estimates per condition
AB for *R^2^* estimate	$R_{\mathrm{est}}^{2}-\text{Mean}(R_{\text{true}}^{2})$	$R_{\mathrm{est}}^{2}$ is the proportion of variance explained in the outcome by the discrepancy estimate, and $R_{\text{true}}^{2}$ is the proportion of variance explained in the outcome by the true discrepancy scores

#### 2.3.1 Bias of discrepancy estimate

First, estimated discrepancy scores were compared to the true discrepancy score to determine accuracy of the discrepancy estimate. To assess accuracy, the absolute bias (AB) for the estimated discrepancy score was calculated as the difference between the true score and the estimate.

AB equal to zero indicated an unbiased estimate of the parameter. A negative AB indicated an underestimation of the parameter (i.e., the estimated value was smaller than the true parameter value), whereas a positive AB indicated an overestimation of the parameter (i.e., the estimated value was larger than the true parameter value).

#### 2.3.2 Reliability of estimates

Reliability of discrepancy estimates was calculated by dividing the variance of the true discrepancy scores by the variance of the discrepancy estimates, as shown in the reliability equation in Table 2. Reliability is normally thought of as ranging from zero to one because the variance of observed scores is normally greater than the variance of true scores. However, because of the EBD shrinkage effect described in Equation 7, the variance of EBD estimates was sometimes less than the variance of true discrepancy scores. This resulted in reliability estimates greater than one. Therefore, in this study, an estimate is most reliable when its reliability is closest to one, indicating that the variance of true scores is equal to variance of estimates. Distance from one, whether in the positive or negative direction, indicated deviance from perfect reliability. Reliability less than one happened when the discrepancy estimate was more variable than the true discrepancy score. Reliability greater than one happened when the discrepancy estimate was less variable than the true discrepancy score.

#### 2.3.3 Predictive power

Although the accuracy of discrepancy estimates is interesting on its own, in practice, it is useful to understand how the estimated discrepancy impacts an outcome. For example, in addition to studying the amount of discrepancy in marital satisfaction, a researcher may also be interested in how the discrepancy predicts an outcome like depression. In this simulation, the discrepancy estimates were used in a regression analysis to predict an outcome. The accuracy of prediction and hypothesis testing was evaluated.

A previously stated, the regression models appropriate for indistinguishable and distinguishable dyads are not the same (see Equations 10, 12, respectively), and this impacts the calculation of discrepancy values. For indistinguishable dyads, only the amount of discrepancy matters, because the direction of discrepancy is arbitrary depending on which dyad member is assigned as *A* and which is assigned as *B*. In this scenario, the absolute value of the discrepancy score can be used as the independent variable in the regression (see Equation 10). Following this, the absolute value of discrepancy estimates (RSD-AV, EBD-AV, and SEM-AV) were each used independently to predict the outcome, and the resulting parameter estimates (i.e., estimated slopes and their standard errors) were assessed for bias.

As previously mentioned regarding distinguishable dyads, the direction of the discrepancy is lost when using the absolute value of the discrepancy as an independent variable, as with the indistinguishable dyad case. Therefore, using the distinguishable dyad equation (Equation 12), the absolute value of discrepancy estimates (RSD-AV, EBD-AV, and SEM-AV), together with W which indicated the direction of the discrepancy, were each used independently to predict the outcome, and the parameter estimates were assessed for bias.

#### 2.3.4 Bias, power, and coverage rates for parameter estimates and standard errors

Bias of parameter estimates demonstrated how accurate discrepancy estimates from each of the three methods predicted the simulated outcome. First, the bias for the parameter estimates (intercept and slopes) and their standard errors were computed as described in the bias equations for parameters and standard errors in Table 2. The bias for standard errors equation in Table 2 shows that because there is no true score standard error to use in the bias calculation, standard error estimates were compared to the standard deviation of all estimates within each simulation condition.

Additionally, the power for the slope estimates was computed as the proportion of statistically significant estimates out of the total number of estimates (see Table 2). The coverage rate was computed as the proportion of cases where the true value of the slope is found within the confidence interval for the slope. Higher coverage rates indicate better estimates of the regression parameters.

Secondly, the accuracy of the *R^2^* estimate was examined by computing the bias, compared with the *R^2^* obtained in the true score regression model. The equation is shown in Table 2.

#### 2.3.5 Impact of design factors on outcomes

Finally, analysis of variance (ANOVA) was used to determine which estimates were most influenced by manipulations of design factors. The corresponding effect sizes (*η*^2^ = SS_effect_/SS_total_) were used to determine the contribution of the five design factors (ICC, cluster number, reliability, effect size, and effect size variance) and method (RSD, EBD, and SEM) to the accuracy of the discrepancy estimation. Post-hoc ANOVAs predicting bias of discrepancy estimation with the five design factors were conducted separately for each of the three methods (RSD, EBD, and SEM), rather than using method as an independent variable, which aided in the interpretation of the impact of design factors on bias of estimates from each method.

## 3 Results

Table 3 includes the means and standard deviations of estimates. Table 3 shows on average how the three methods compared on all evaluation measures. For example, the first row of Table 3 shows that average bias of discrepancy estimates was zero for all three methods. Table 4 shows the effect sizes (*η*^2^) for all six-way ANOVAs measuring the impact of the design factors (method, reliability, ICC, cluster number, effect size, and effect size variance) and all two-way interactions on the measures of accuracy for the three estimation methods. Table 4 is important for understanding which measures were substantially impacted by variation in study conditions. Table 5 includes effect sizes (*η*^2^) for post-hoc five-way ANOVAs measuring the impact of the design factors (reliability, ICC, cluster number, effect size, and effect size variance) and all two-way interactions on the measures of accuracy for the three estimation methods individually. In Tables 4, 5, only effect sizes at least 0.01 are shown in the table, and only medium and large effects of 0.06 or greater (per Cohen, 1988) are interpreted and discussed.

**Table 3:** Mean (standard deviation) of bias, reliability, and discrepancy as predictor estimates.

Estimate	Estimate property	RSD	EBD	SEM
Accuracy of discrepancy	Absolute bias (AB) of discrepancy	0.00 (0.06)	0.00 (0.06)	0.00 (0.13)
	Reliability of discrepancy	0.51 (0.06)	2.29 (63.70)	0.53 (0.06)
Discrepancy as predictor				
Discrepancy slope	AB of estimate	−0.26 (0.11)	0.22 (0.43)	−0.25 (0.12)
	AB of standard error	0.00 (0.02)	−0.07 (0.28)	0.00 (0.02)
	Power of estimate	0.96 (0.20)	0.95 (0.22)	0.96 (0.20)
	Coverage rate of estimate	0.91 (0.29)	0.24 (0.43)	0.89 (0.31)
Direction slope	AB of estimate	−0.29 (0.32)	−0.29 (0.38)	−0.29 (0.35)
	AB of standard error	0.00 (0.04)	−0.03 (0.12)	−0.02 (0.04)
	Power of estimate	0.14 (0.35)	0.16 (0.36)	0.16 (0.37)
	Coverage rate of estimate	0.77 (0.42)	0.75 (0.43)	1.00 (0.00)
Discrepancy by direction interaction slope	AB of estimate	−0.13 (0.16)	−0.08 (0.58)	−0.13 (0.16)
	AB of standard error	0.00 (0.02)	−0.07 (0.41)	0.00 (0.02)
	Power of estimate	0.09 (0.29)	0.09 (0.29)	0.10 (0.29)
	Coverage rate of estimate	0.18 (0.39)	0.58 (0.49)	0.19 (0.40)
R-squared	AB of estimate	−0.04 (0.03)	−0.05 (0.04)	−0.04 (0.03)
	Power of estimate	0.99 (0.09)	0.98 (0.13)	0.99 (0.09)

**Table 4:** Summary of *η*^2^ for the six-way ANOVA main and first-order interaction effects (*η*^2^ ≥ 0.01).

	Discrepancy		Slope—discrepancy		Slope—direction		Slope—discrepancy * direction interaction		*R* ^2^
	Estimate bias	Reliability of estimate	Estimate bias	Standard error bias	Estimate bias	Standard error bias	Estimate bias	Standard error bias	Estimate bias
Method	–	–	0.42	0.05	–	0.04	0.01	0.02	0.69
N	–	–	–	0.03	–	0.03	–	0.03	–
ICC	–	–	–	0.01	–	–	–	0.01	–
Reliability	–	–	–	–	–	–	–	–	–
Effect size	–	–	–	–	–	0.01	–	–	0.01
Effect size variance	–	–	–	0.01	–	–	–	–	0.02
M*N	–	–	–	0.06	–	0.02	–	0.05	0.00
M*I	–	–	0.02	0.02	–	0.01	–	0.02	0.01
M*R	–	–	–	–	–	–	–	–	–
M*ES	–	–	–	0.01	–	–	–	0.01	–
M*ESV	–	–	0.01	0.01	–	0.01	–	0.01	0.01
N*I	–	–	–	0.02	–	–	–	0.02	–
N*R	–	–	–	–	–	–	–	–	–
N*ES	–	–	–	0.01	–	–	–	0.01	–
N*ESV	–	–	–	0.01	–	–	–	0.01	–
I*R	–	–	–	–	–	–	–	–	–
I*ES	–	–	–	–	–	–	–	–	–
I*ESV	–	–	–	0.01	–	–	–	–	–
R*ES	–	–	–	–	–	–	–	–	–
R*ESV	–	–	–	–	–	–	–	–	–
ES*ESV	–	–	–	–	–	–	–	–	–

Design factors are abbreviated in the table as (a) N = cluster number; (b) M = method; (c) I = ICC; (d) R = reliability; (e) ES = effect size; (f) ESV = effect size variance. Bias refers to the absolute bias (AB) defined in Table 2.

**Table 5:** Summary of *η*^2^ for the post-hoc five-way ANOVA main and first order interaction effects (*η*^2^ ≥ 0.01).

	Reliability			Discrepancy slope bias			Discrepancy slope standard error bias			Direction slop standard error bias			Interaction slope standard error bias			*R*^2^ bias		
	RSD	EBD	SEM	RSD	EBD	SEM	RSD	EBD	SEM	RSD	EBD	SEM	RSD	EBD	SEM	RSD	EBD	SEM
N	–	–	0.02	–	–	–	–	0.09	–	–	0.05	0.06	–	0.08	0.01	–	0.02	0.01
ICC	0.06	–	0.04	0.02	0.03	0.02	–	0.04	–	–	0.01	0.03	–	0.03	–	0.03	0.04	0.02
Reliability	–	–	–	–	–	–	–	–	–	–	–	–	–	–	–	–	–	–
Effect size	0.24	–	0.23	0.01	0.01	–	–	0.01	–	–	0.01	0.01	–	0.01	–	0.05	0.02	0.04
Effect size variance	0.55	–	0.52	0.02	0.02	0.02	–	0.02	–	–	0.01	–	–	0.01	–	0.11	0.05	0.07
N*I	–	–	–	–	–	–	–	0.06	–	–	0.02	–	–	0.06	–	–	–	–
N*R	–	–	–	–	–	–	–	–	–	–	0.01	–	–	–	–	–	–	–
N*ES	–	–	–	–	–	–	–	0.02	–	–	0.02	–	–	0.03	–	–	–	–
N*ESV	–	–	–	–	–	–	–	0.03	–	–	0.01	–	–	0.03	–	–	–	–
I*R	–	–	–	–	–	–	–	–	–	–	–	–	–	–	–	–	–	–
I*ES	0.01	–	0.01	–	–	–	–	0.01	–	–	0.01	–	–	0.01	–	–	–	–
I*ESV	0.01	–	0.01	–	–	–	–	0.02	–	–	–	–	–	0.01	–	–	–	–
R*ES	–	–	–	–	–	–	–	–	–	–	–	–	–	–	–	–	–	–
R*ESV	–	–	–	–	–	–	–	–	–	–	–	–	–	–	–	–	–	–
ES*ESV	0.01	–	0.01	–	–	–	–	0.01	–	–	0.01	–	–	–	–	–	–	–

Post-hoc ANOVAs were conducted for each method separately to aid in the interpretation of the effects. The dependent variables were discrepancy bias, reliability of discrepancy, three bias slope estimates and their standard errors (discrepancy, direction, and interaction), and R^2^ bias. The dependent variables discrepancy bias, direction slope, and interaction slope are excluded from the table because design factors were not substantial predictors (*η*^2^ ≥ 0.01) for any of the 3 methods. All other dependent variables are included in columns above. Design factors are abbreviated in the table as (a) N = cluster number; (b) M = method; (c) I = ICC; (d) R = reliability; (e) ES = effect size; (f) ESV = effect size variance.

### 3.1 Bias of discrepancy estimates

The average bias of all three discrepancy estimates was zero. Furthermore, ANOVA results showed no notable effect sizes using method and design factors to explain the bias of discrepancy estimates. In other words, no variations of method or design factors accounted for a significant amount of bias in discrepancy estimates.

### 3.2 Reliability of discrepancy estimates

As described in the methods, reliability is a measure of the consistency of the discrepancy estimates. Perfect reliability occurred when the variance of discrepancy estimates is equal to the variance of true discrepancy score, resulting in reliability equal to one. Estimates were considered less reliable as reliability values deviated further from one. On average, reliability values of EBD estimates were the furthest from one and therefore the least reliable (reliability = 2.29, *σ* = 63.70). Reliability for RSD (reliability = 0.51, *σ* = 0.06) and SEM (reliability = 0.53, *σ* = 0.06) were better. Furthermore, initial six-way ANOVA results showed no substantial effect sizes using method and design factors to explain the reliability of discrepancy estimates. However, some effects were found using the post-hoc ANOVAs described below.

**Figure 2:**
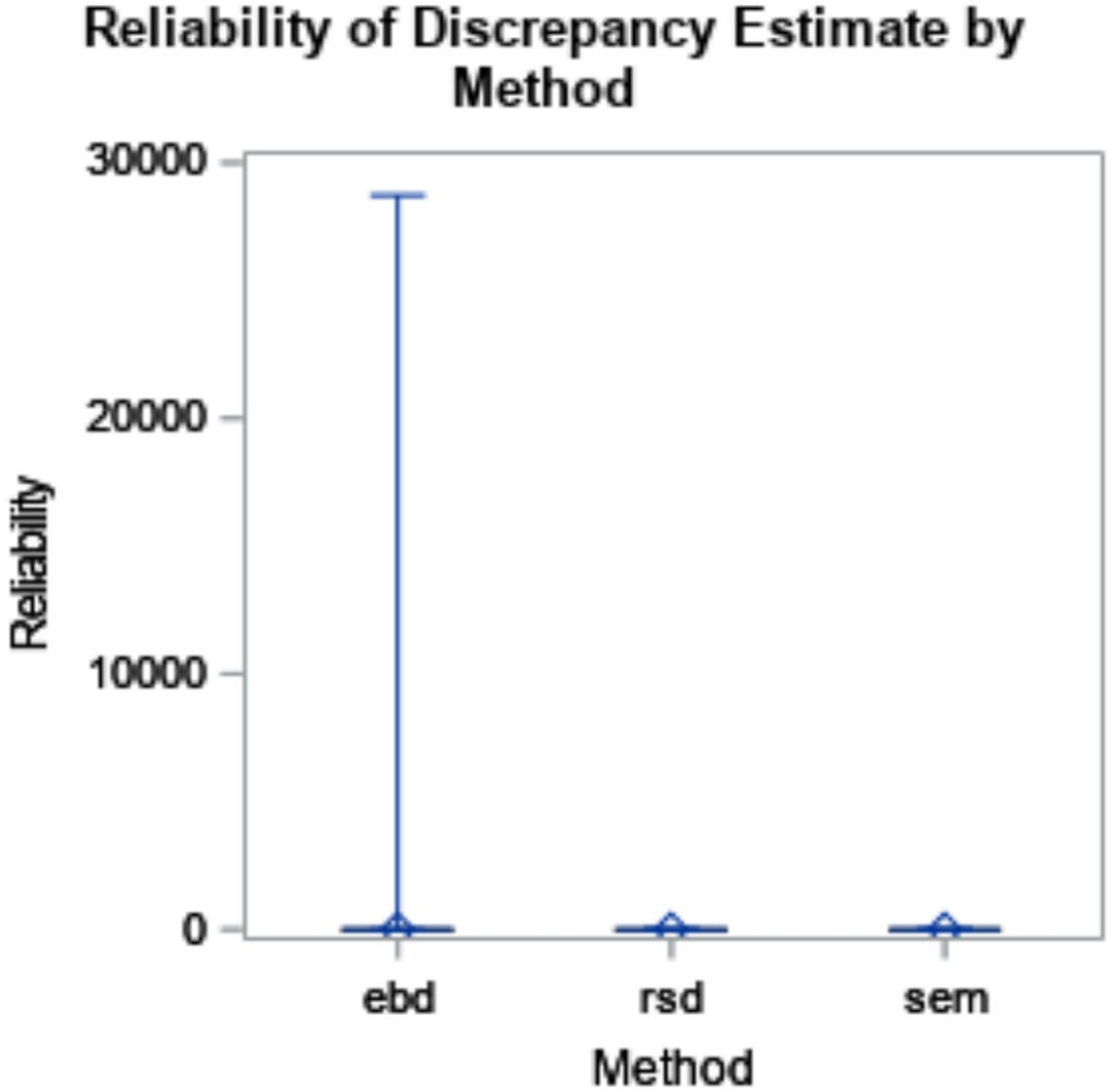
Reliability of discrepancy estimate by method (*η*^2^ < 0.001). The minimum and maximum bias are represented by the endpoints of each plot. The upper edge of the box represents the third quartile (75th percentile), and the lower edge of the box represents the first quartile (25th percentile). The median (50th percentile) is represented by the line within the box, and the mean is represented by the diamond within the box.

The six-way ANOVA results showed that reliability was not substantially impacted by method or design factors. Though the means did not substantially differ by method, the box plot in Figure 2 shows the range for EBD is impacted by outliers.

#### 3.2.1 Post-hoc ANOVAs by method

**Figure 3:**
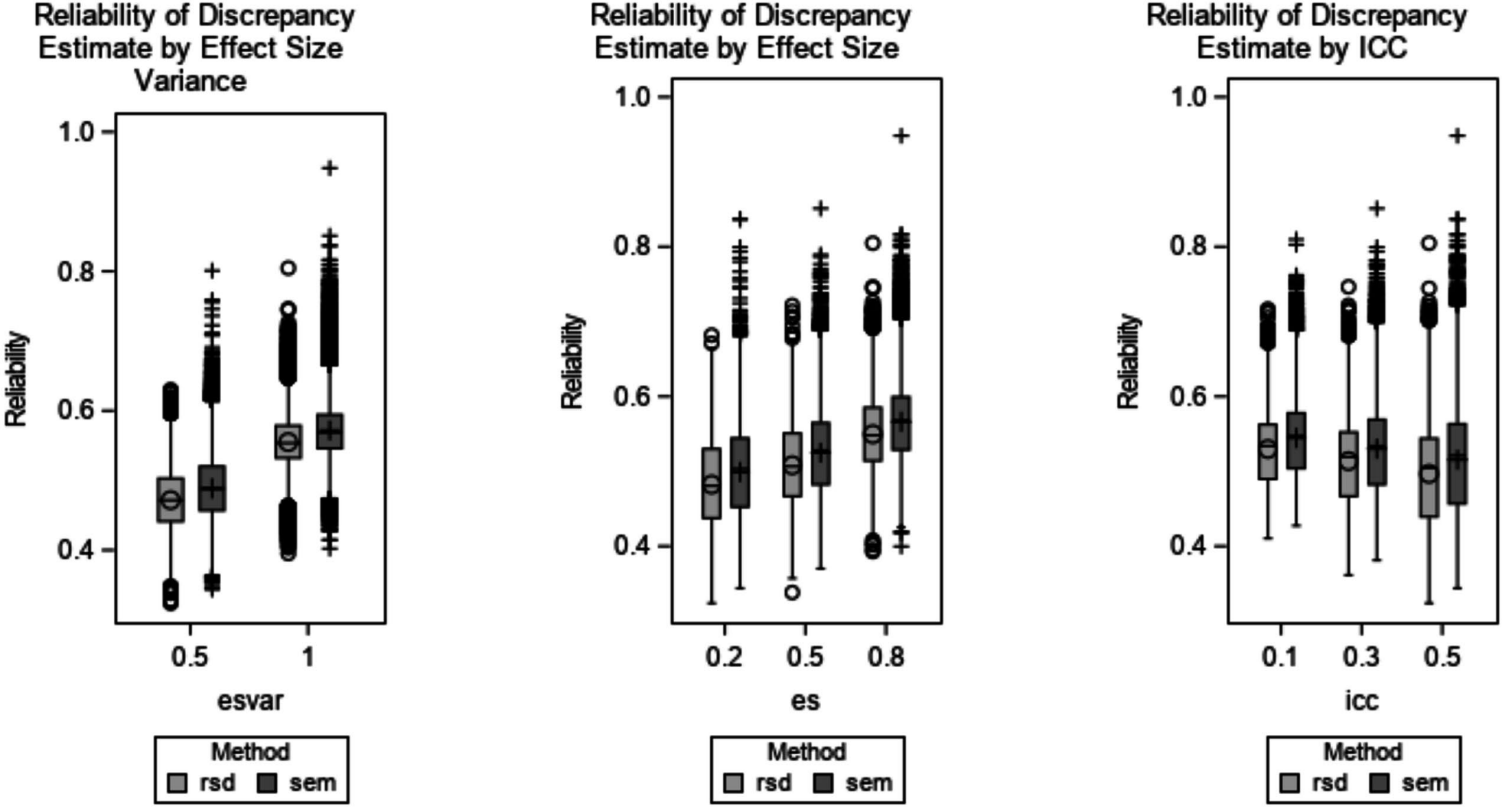
Reliability of RSD and SEM discrepancy estimate by design factors. Design factors shown are effect size variance (RSD *η*^2^ = 0.55 and SEM *η*^2^ = 0.52), effect size (RSD *η*^2^ = 0.24 and SEM *η*^2^ = 0.23), and ICC (RSD *η*^2^ = 0.06 and SEM *η*^2^ = 0.04). EBD is excluded because *η*^2^ < 0.01 for all effects. The minimum and maximum bias are represented by the ends of each plot. The upper edge of the box represents the third quartile (75th percentile), and the lower edge of the box represents the first quartile (25th percentile). The median (50th percentile) is represented by the line within the box, and the mean is represented by the symbol within the box. Outliers are labeled with the O symbol for RSD and the + symbol for SEM.

Post-hoc ANOVAs were conducted for each method individually. As shown in Table 5, reliability of EBD estimates was not substantially (*η*^2^ > = 0.01) impacted by any design factor. RBD and SEM reliability were each greatly impacted by effect size variance (RSD *η*^2^ = 0.55 and SEM *η*^2^ = 0.52) and effect size (RSD *η*^2^ = 0.24 and SEM *η*^2^ = 0.23). To a lesser extent, RBD and SEM reliability were impacted by ICC (RSD *η*^2^ = 0.06 and SEM *η*^2^ = 0.04). Reliability increased as effect size variance and effect size increased. Reliability decreased as ICC increased. Across all levels of design factors, SEM reliability was greater than RSD. These trends are further illustrated in Figure 3.

### 3.3 Predictive power of discrepancy estimates

Simulated regression models were used to evaluate the accuracy of discrepancy estimation methods in predicting an outcome. There were three slope estimates in the regression model: (1) discrepancy slope, which was the slope estimate for the discrepancy; (2) direction slope, which was the slope estimate for the dichotomous indicator of direction of discrepancy (i.e., 0 where the score from dyad member A is greater than the score from dyad member B, and 1 where the score from dyad member A is less than the score from dyad member B), and (3) slope (discrepancy by direction interaction), representing the interaction effect between discrepancy and direction.

Results presented below are for distinguishable dyads. The prediction model for indistinguishable dyads, which only included one discrepancy slope, had similar results as the discrepancy slope for distinguishable dyads, rendering those results redundant. For each of the three slope estimates as well as *R^2^* estimates, the results include descriptive statistics for bias, ANOVA results, power, and coverage rate.

#### 3.3.1 Discrepancy slope estimate and standard error bias

**Figure 4:**
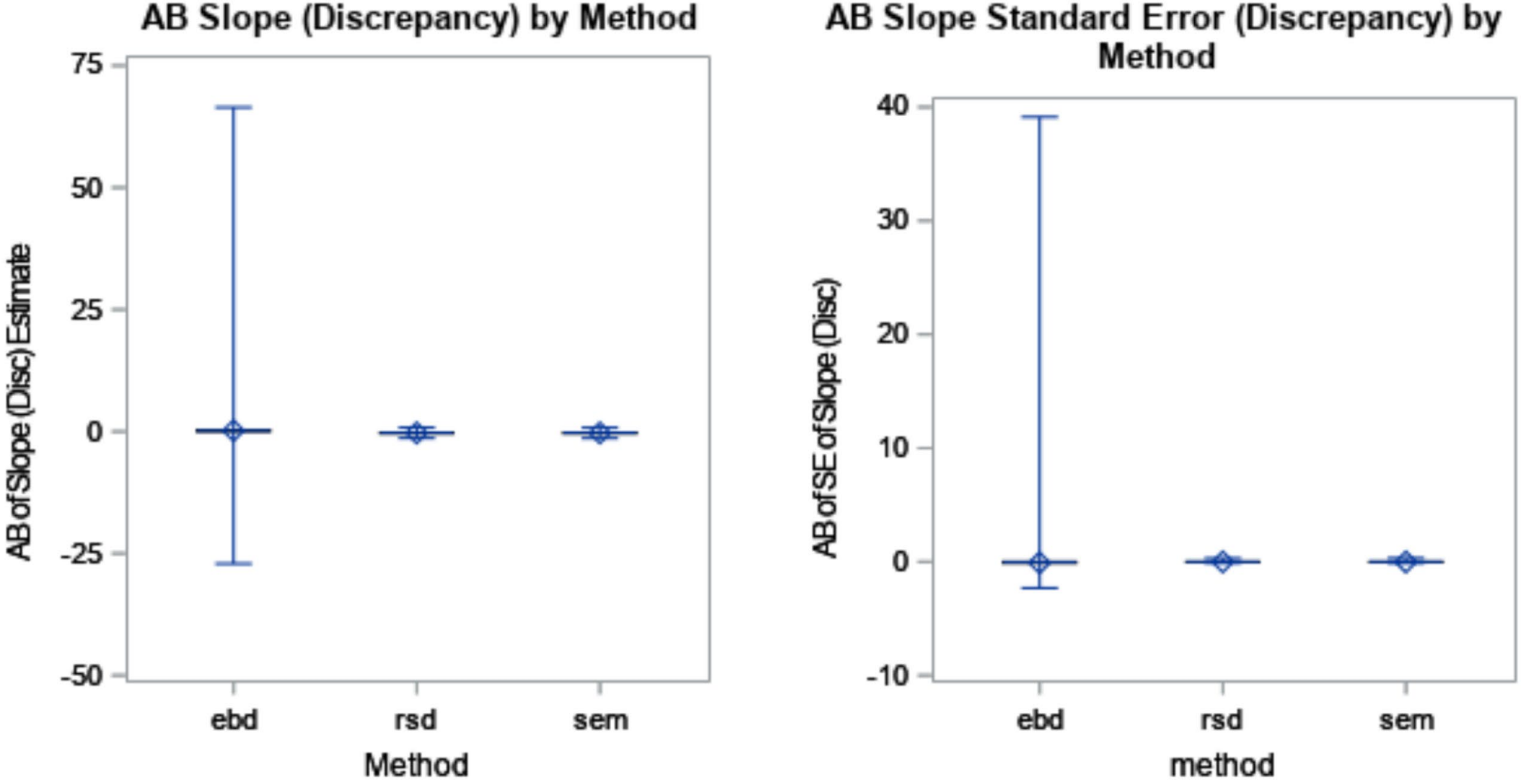
Absolute bias (AB) of discrepancy slope estimate by method (*η*^2^ = 0.42) and AB of standard error (SE) of discrepancy slope estimate by method (*η*^2^ = 0.05). The minimum and maximum bias are represented by the endpoints of each plot. The upper edge of the box represents the third quartile (75th percentile), and the lower edge of the box represents the first quartile (25th percentile). The median (50th percentile) is represented by the line within the box, and the mean is represented by the diamond within the box.

The discrepancy slope estimates, estimating the strength of the relationship between the discrepancy and the outcome, were slightly less biased for EBD (AB = 0.22) than RSD (AB = 0.26) and SEM (AB = 0.25). However, the range of AB for EBD (min AB = −27.1, max AB = 66.5) was vastly larger that of RSD and SEM, which both ranged about −1.4 to −0.3. The standard deviation of AB for EBD was 0.43. The large range in conjunction with the relatively reasonable standard deviation indicated that AB of slope estimate (discrepancy) for EBD included extreme outliers (i.e., an outlier exceeding 3*interquartile range below the 1st quartile or above the 3rd quartile). The boxplots shown in Figure 4 illustrate the distributions of bias by method. Differences among methods, as shown in Figure 4, accounted for a substantial amount of variance in bias of discrepancy slope estimate (*η*^2^ = 0.42).

Similarly, the standard errors of slope estimates in the regression equation were most biased for the EBD method (AB = 0.08), and on average, equal to zero for RSD and SEM. This means that the estimates of the relationship between discrepancy and the outcome were less consistent for the EBD method, and quite consistent for RSD and SEM. In the ANOVA, differences among methods accounted for an *η*^2^ of 0.05.

**Figure 5:**
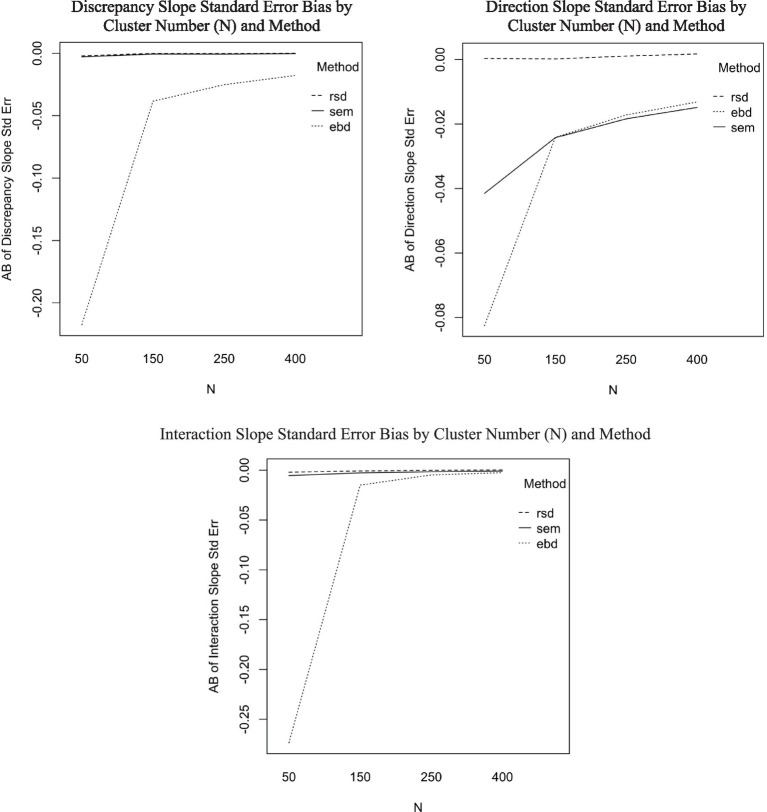
Standard error bias by N and method for discrepancy slope, direction slope, and interaction slope.

**Figure 6:**
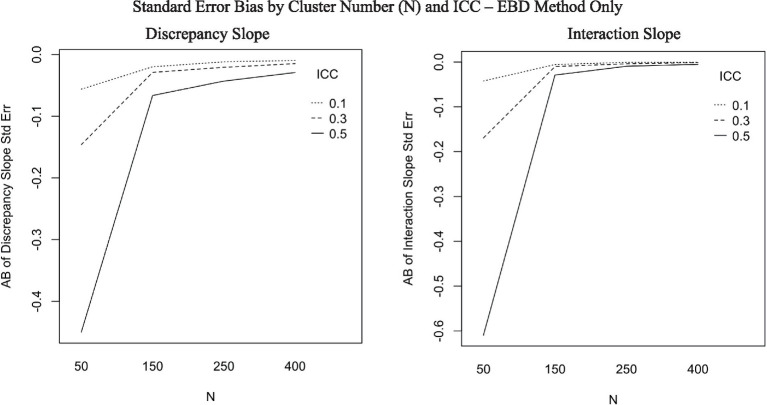
Absolute bias (AB) of discrepancy slope and interaction slope standard error by cluster number (N) and ICC, for EBD method only (*η*^2^ = 0.06 for each plot).

The ANOVA predicting AB of standard error estimates for discrepancy slope showed one interaction with effect size of at least 0.06: the interaction between method and N-size (*η*^2^ = 0.06). Post-hoc ANOVAs were conducted separately for each method to aid in interpretation of the six-way ANOVAs. Figure 5 includes three plots showing substantial effects of cluster number (N) on standard error of slope estimates for the EBD method. The first plot in Figure 5, for discrepancy slope standard error bias, shows that standard error bias is stable across cluster number for RSD and SEM methods, but increases as cluster number increases for EBD (*η*^2^ = 0.09). Furthermore, cluster number interacted with ICC for the EBD method (*η*^2^ = 0.06). The interaction effect, plotted in Figure 6, shows that the effect of cluster number (N) on AB of discrepancy slope standard errors increases as ICC increases.

The coverage rate (i.e., the percentage of models in which the true score discrepancy slope was found in the confidence interval for the discrepancy slope estimates) was larger for the RSD (91%) and SEM (89%) methods, while EBD was 24%. This indicates that the RSD and SEM methods more accurately predicted the strength of the relationship between the discrepancy and the outcome than EBD.

The power (aka proportion of statistically significant estimates) for the discrepancy slope estimate was computed for the three estimation methods. The power for the RSD and SEM was 0.96, and for EBD, 0.95.

#### 3.3.2 Direction slope estimate and standard error bias

**Figure 7:**
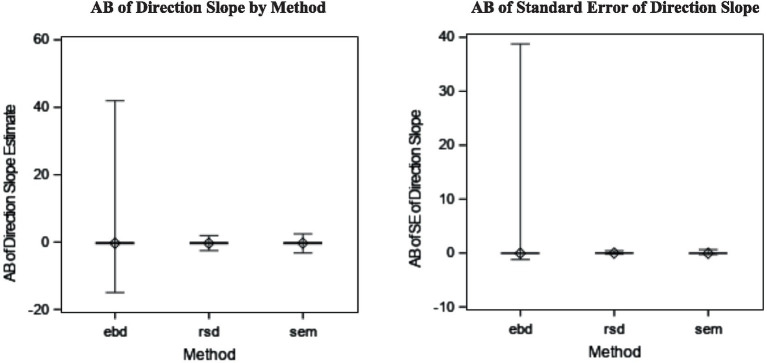
Absolute bias (AB) of direction slope estimate by method (*η*^2^ = 0.42) and AB of standard error (SE) of direction slope estimate by method (*η*^2^ = 0.05).

The bias of direction slope estimates was the same for all three methods (AB = −0.29). That means the direction of the discrepancy for distinguishable dyads’ relationship with the outcome was estimated with similar levels of bias for all three methods. However, the EBD method suffers from outliers, shown in the boxplots in Figure 7.

The average bias of standard errors was also comparable among all three methods, with RSD being the least biased (AB = 0.00), followed by SEM (AB = −0.03) and EBD (AB = −0.03). The boxplots of these distributions are shown in Figure 5 to illustrate EBD’s outliers. Neither the slope estimate nor its standard error were substantially (*η*^2^ > = 0.06) impacted by method and design factors in the original ANOVAs (see Table 4). However, the post-hoc ANOVAs revealed that standard error bias from the SEM and EBD methods was substantially impacted by cluster number, as shown in the upper-right plot in Figure 5. Standard error bias decreased as cluster number increased for EBD (*η*^2^ = 0.05) and SEM (*η*^2^ = 0.06).

The coverage rate of the direction slope estimate was 100% for SEM and lower for RSD (77%) and EBD (75%). The power for direction slope estimate was quite low among all methods, ranging from 0.14 for RSD to 0.16 for EBD and SEM. Lower power was expected because the outcome variable was generated based on a true slope of 0.5. It makes sense that power is lower for direction slope than discrepancy slope, which was generated based on a true slope of 0.8. A stronger relationship between outcome and predictor results in a greater percentage of statistically significant results.

#### 3.3.3 Discrepancy by direction interaction slope estimate and standard error bias

**Figure 8:**
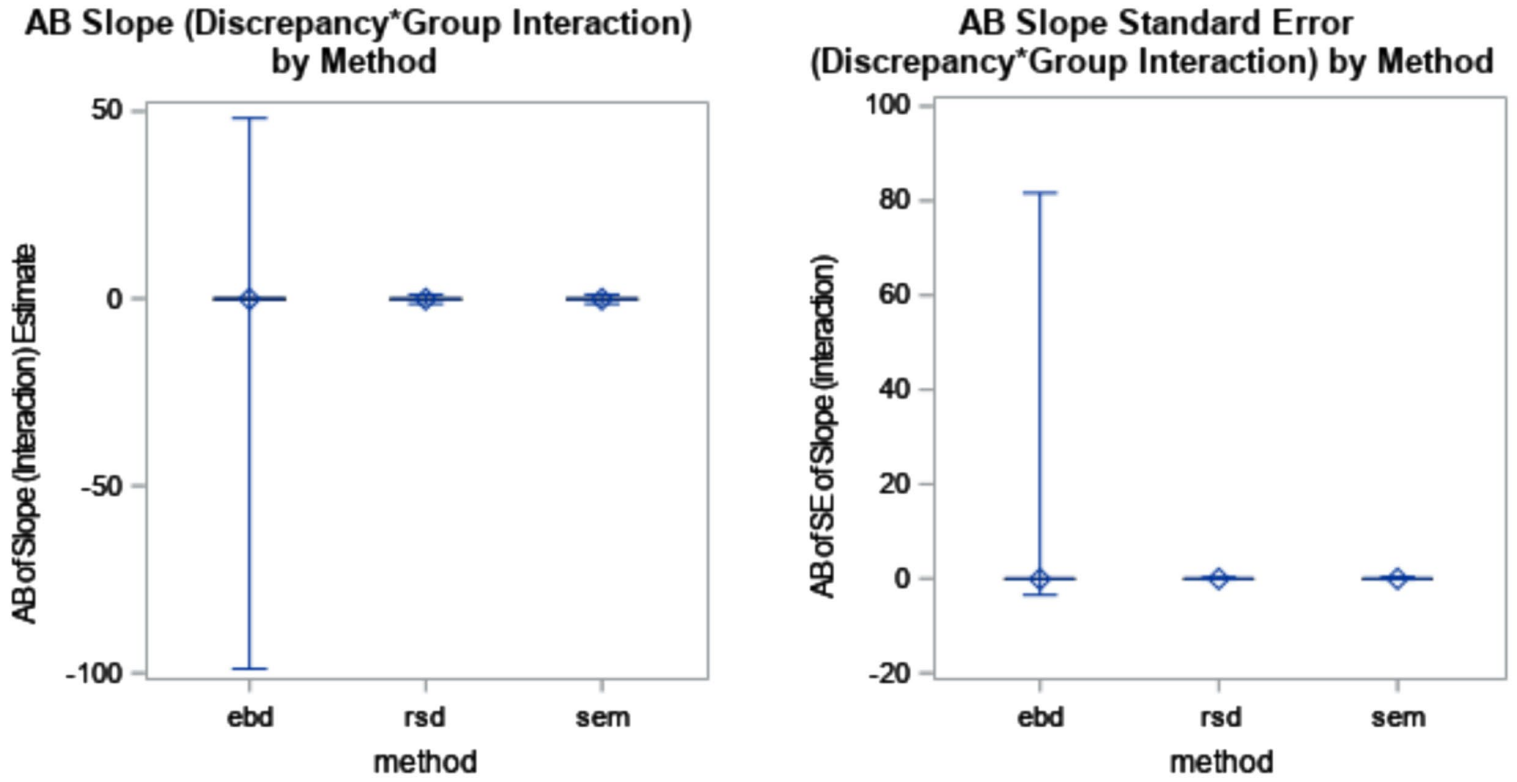
Absolute bias (AB) of interaction slope estimate by method (*η*^2^ = 0.42) and AB of standard error (SE) of interaction slope estimate by method (*η*^2^ = 0.05).

The AB of the discrepancy by slope interaction effect was the greatest for RSD and SEM, both with AB = −0.13. EBD had bias of −0.08. Outliers remained prevalent for the EBD method. The distributions are shown in Figure 8.

The mean AB of standard errors was 0 for RSD and SEM, and −0.07 for EBD. The EBD method produced extreme standard error outliers, with standard error bias ranging from −3.46 to 81.63. Neither the slope estimate nor its standard error were substantially (*η*^2^ > = 0.06) impacted by method and design factors in the ANOVAs (see Table 4). According to the post-hoc ANOVAs, however, the RSD and SEM methods were not substantially impacted by design factors, but the EBD method was. As shown in the bottom plot of Figure 5, bias decreased sharply as cluster number (N) increased. Furthermore, cluster number interacted with ICC for the EBD method. Figure 6 shows that the effect of cluster number on standard error bias increases as ICC increases.

The mean AB of standard errors was 0 for RSD and SEM, and −0.07 for EBD. The EBD method produced extreme standard error outliers, with standard error bias ranging from −3.46 to 81.63. Neither the slope estimate nor its standard error were substantially (*η*^2^ > = 0.06) impacted by method and design factors in the ANOVAs (see Table 4). According to the post-hoc ANOVAs, however, the RSD and SEM methods were not substantially impacted by design factors, but the EBD method was. As shown in the bottom plot of Figure 5, bias decreased sharply as cluster number (N) increased. Furthermore, cluster number interacted with ICC for the EBD method. Figure 5 shows that the effect of cluster number on standard error bias increases as ICC increases.

The coverage rate of discrepancy*direction interaction slope estimate was highest for EBD (58%). It was substantially lower for SEM (19%) and RSD (18%). Finally, the power of the discrepancy*direction interaction estimates was low and comparable among the three methods. Power was highest for SEM (0.10), followed by RSD (0.09) and EBD (0.09).

#### 3.3.4 *R^2^* bias

**Figure 9:**
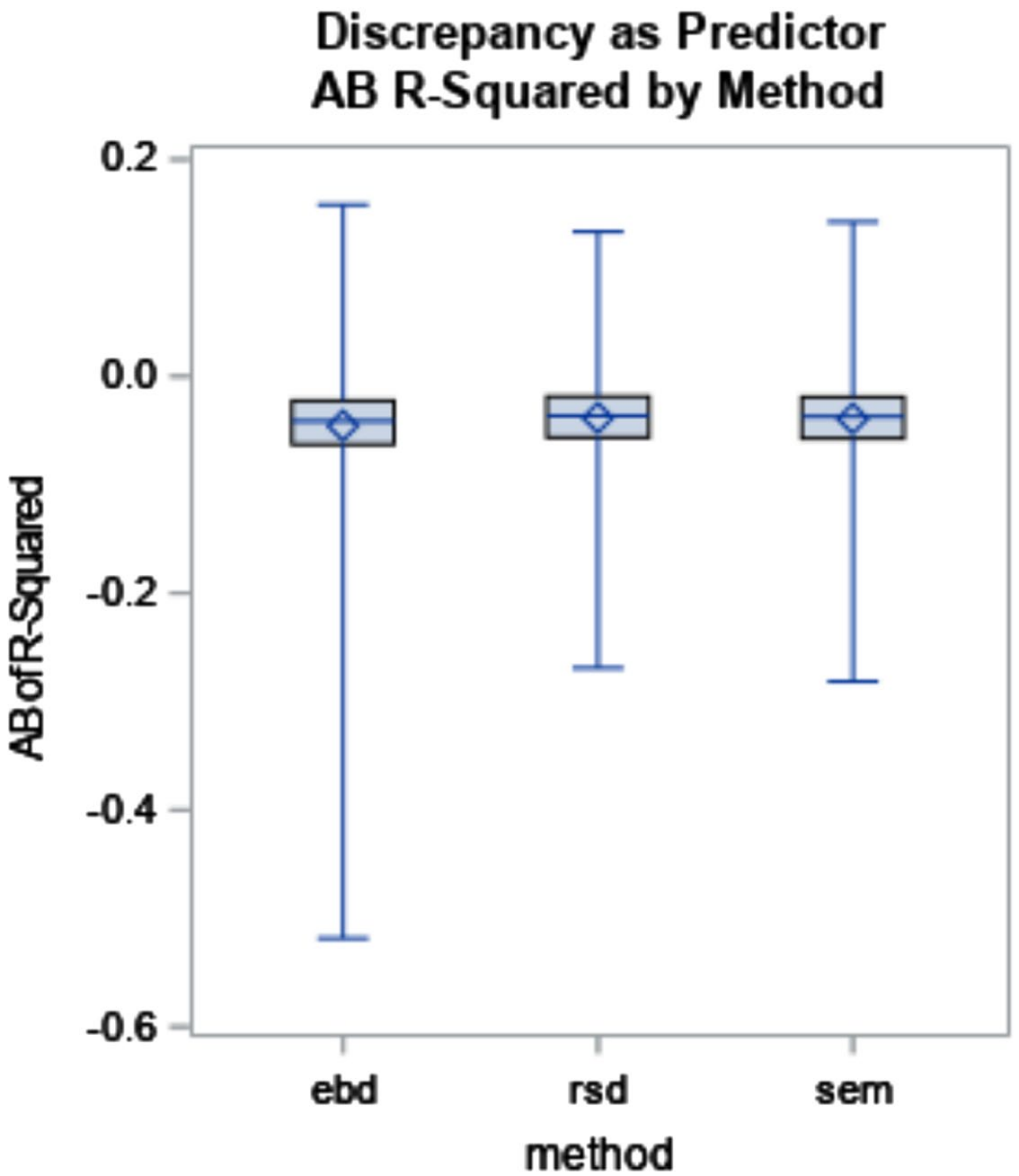
Absolute bias (AB) of *R*^2^ by method (*η*^2^ = 0.69).

**Figure 10:**
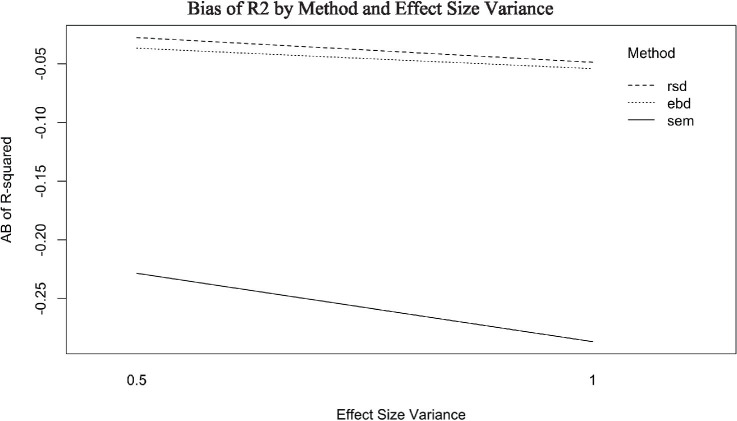
Absolute bias (AB) of *R*^2^ by method and effect size variance.

RSD and SEM *R^2^* bias values were −0.04, and EBD was −0.05. Estimation method explained a large proportion of the variance in *R*^2^ bias (*η*^2^ = 0.69). EBD accounted for the differences among methods as shown in Figure 9. The post-hoc ANOVAs explained that *R^2^* bias from each method (RSD, EBD, and SEM) was substantially impacted by effect size variance. Bias by method and effect size variance is plotted in Figure 10. The plot shows that *R^2^* bias increases as effect size variance increases. The power of *R*^2^ was high, at 0.98 for EBD, and 0.99 for RSD and SEM. The high power of *R*^2^ values across all three methods indicated that each method produced a statistically significant *R*^2^, effectively identifying the existence of a relationship between the outcome and predictors.

## 4 Discussion

These findings suggest that dyadic discrepancy from MLM (i.e., EBD) suffers from poor reliability, especially where ICC was high, effect size variance was high, and cluster number was low, which hinders its accuracy as a predictor. The implications of this finding are that RSD or SEM may be preferred because they are not impacted as greatly by ICC, effect size variance, and cluster number. These findings are discussed more fully below, followed by a discussion of limitations and recommendations for future research.

### 4.1 Why did the EBD shrinkage effect result in outliers?

**Figure 11:**
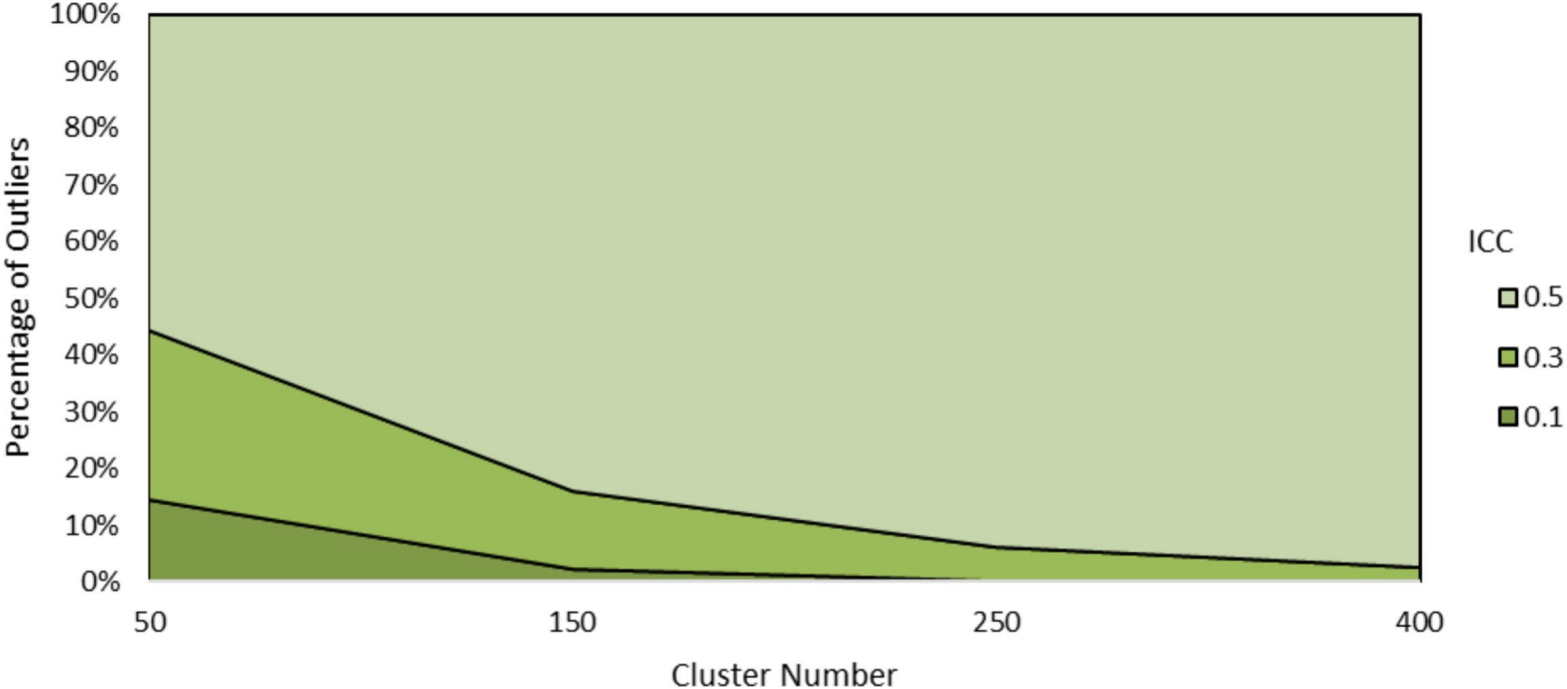
Percentage of outliers by ICC and cluster number.

The data were explored further to better understand why EBD produced outliers in prediction. First “outlier” conditions were defined as those with AB discrepancy slope for EBD fell beyond the range of AB discrepancy slope for RSD and SEM, which was about −1.4 to −0.8. A total of 8,111 (or 3.8%) of AB of EBD estimates met this condition. Outliers were most prevalent where ICC equaled 0.5 and cluster number equaled 50, as shown in Figure 11.

Examining the outlier data showed that variance of EBD within outlier samples was lower than the non-outlier samples. This led to a deeper analysis of the variance components in EBD estimation. According to Equation 7, EBD is a weighted combination of the OLS slope and the grand mean of slopes across all dyads (γ_10_). It is weighted by the reliability of the OLS estimate. The more concentrated β_1j_ values are around γ_10_, the more γ_10_ is weighted in EBD. The more the central tendency is weighted in EBD, the less variable EBD will be. In extreme outlying cases, the variance of EBD was near zero, indicating over shrinkage, which was cautioned against by Raudenbush (2008), particularly when cluster sizes are small. As a result, EBD became ineffective as a predictor in the regression models.

### 4.2 Which method should researchers use to estimate dyadic discrepancy?

One purpose of this study was to determine the most accurate method for estimating dyadic discrepancy. On average, AB was zero for all three methods evaluated (RSD, EBD, and SEM). Using method and design factors to predict bias in an ANOVA resulted in *η*^2^ less than 0.0001. Considering average bias alone, one might conclude that all three methods are equally accurate.

However, though the methods do not differ in average bias, they do differ in reliability of bias. The EBD method produced estimates with the poorest reliability. On average, reliability was 2.29; being greater than one indicates that variance of EBD estimates was less than variance of true discrepancy scores. This proved to be problematic for bias of estimates and their standard errors in prediction, as discussed below.

Another purpose of the study was to determine which method’s discrepancy estimates most accurately predict an outcome. Prediction was evaluated using slope estimate bias, standard error of slope estimate bias, power, coverage rate, and effect size (*R*^2^). The discrepancy estimates from the EBD method produced extreme outliers in the prediction models. This resulted in EBD being the poorest performer by far in slope estimate bias and standard error bias for all three slopes (discrepancy, direction, and discrepancy*direction interaction). ANOVA effect size, *η*^2^, for method was not large except for when predicting discrepancy slope bias (*η*^2^ = 0.42). Small effects were seen using method to predict bias of slope standard error (slope discrepancy S.E. *η*^2^ = 0.05, direction = 0.04, and discrepancy*direction interaction = 0.02), as well as slope (interaction) bias (*η*^2^ = 0.01).

The issues with EBD stem from the “over-shrinkage” described in the literature review above (Raudenbush, 2008; Raudenbush and Bryk, 2002; Stage, 2001). In the current study, the over-shrinkage primarily impacted samples with small cluster numbers and, more so, high conditional ICC. Cluster number interacts with method (*η*^2^ = 0.06), and the interaction plots (Figure 5) show that bias was greatest for EBD where cluster size was 50.

An examination of EBD outliers revealed that over 85% of the outlying samples had the high conditional ICC value of 0.5. The variance of EBD in these samples was very low. Because the EBD method partials out the variance from the grand slope (see Equations 3a–5), if dyads have a high level of dependence, more of the variance overall is partialed out from the dyad-level discrepancy. This results in EBD estimates with low variance, because the variance accounted for by the grand slope has been removed already. The shrunken variances of EBD relative to the true score discrepancy caused the extreme outliers in bias of parameters and their standard errors in the prediction equations.

Overall, none of the three methods were clearly more accurate than the other, and bias was not substantially impacted by method or design factor. However, the poor reliability of EBD and the resulting impact on accuracy of predicting an outcome suggests RSD and SEM are better estimates of dyadic discrepancy. RSD may be preferred since it is easier to compute. However, SEM has the advantages of model flexibility, such as predicting the outcome directly in the same model or including other relationships and variables in the model.

### 4.3 Was raw-score difference impacted by reliability?

According to the reliability formula in Equation 1 from Williams and Zimmerman (1977), RSD reliability would be higher when the reliability of scores from individual dyad members was higher and when ICC was smaller. According to Table 5 and Figure 3, the ANOVA predicting RSD reliability with design factors confirmed that reliability for RSD was higher when ICC was lower (*η*^2^ = 0.06). Reliability of scores for dyad members *A* and *B* was expected to have an impact on reliability of discrepancy estimates. However, in the ANOVA predicting reliability of estimates, *η*^2^ was 0 for reliability, indicating that RSD reliability did not depend on the reliability of individual dyad member scores. The reliability values simulated in this study were set high at 0.7, 0.8, and mixed 0.7 and 0.8, per values commonly found in applied research. Future research should include lower levels of reliability as a design factor to test the limits of how low reliability of scores from dyad member *A* and *B* can go without impacting the reliability of discrepancy.

### 4.4 Limitations and recommendations for future research

As explained in the methods and results, reliability is typically thought of as ranging from zero to one, but the EBD shrinkage effect often resulted in reliability values greater than one. We did not identify an alternate method of computing reliability for EBD other than the traditional true score variance divided by observed score variance. Future research should consider whether there is a better way to compute or interpret the reliability of EBD.

Furthermore, one of the benefits of the EBD shrinkage effect is the ability to include dyads with missing or unreliable data, which then “borrow” strength from the rest of the sample (Kim et al., 2013). However, missing data was not considered in this simulation. Varying degrees of missingness could be simulated in future research to generate understanding of whether and how each method overcomes the limitations of missing data.

The findings suggest that even though RSD and SEM had poor reliability, these estimates still do a good job predicting an outcome as evidenced by high levels of power. This suggests that RSD is suitable for many practical, real-life applications of dyadic discrepancy research, despite historical concerns that it is unreliable. Statistically, the reliability of the discrepancy score is impacted by the reliability of each dyad member’s scores, but the conditions prevalent in dyadic data literature may overcome these concerns by having high reliabilities of dyad member’s scores and low to moderate dependence. However, future research should further push the upper and lower limits of the design factors in the current study, specifically reliability of dyad member’s scores and cluster number, in an effort to determine under what conditions the RSD would become unacceptable.

A simple regression model was used to evaluate accuracy of prediction, but in reality, more complicated models are needed to adequately address dyadic discrepancy research questions. While RSD is the most straightforward computational approach, the ability to generate an accurate dyadic discrepancy in SEM is promising for researchers seeking to use the discrepancy in more complicated models, such as the second-order factor model from Newsom (2002). The discrepancy can be generated in the same model as the prediction model, and the measurement model could be included. Though it is possible to output the idiographic discrepancy using SEM, in practice, RSD is easier to compute for purposes of idiographic research. But SEM offers the flexibility of using discrepancy in a nomothetic model while maintaining the ability to output it ideographically as well. Future research should ensure the good performance of the SEM discrepancy in this study is maintained in more complicated models, including those with the measurement model included. It would also be useful to understand the impacts of measurement invariance on dyadic discrepancy and its use in prediction (Russell et al., 2016).

EBD and SEM discrepancy estimates were generated using full-information maximum likelihood (FIML) estimation method. Restricted maximum likelihood (REML) is an alternative estimation method that may result in less biased estimates, particularly when cluster number is small (Raudenbush and Bryk, 2002). Future research should investigate in more detail whether the estimation method matters in the current context.

Finally, the findings here cannot be generalized to conditions not included in the simulation. Furthermore, future research should apply the techniques to real data, as opposed to simulated data, to see if the estimates compare to those found in this study.

### 4.5 Practical implications

Researchers seeking to make an informed decision about which method to use to study dyadic discrepancy would hope for a clear answer about which method is best. Notwithstanding the limitations and directions for future research in the preceding section, the findings suggest that RSD or SEM perform quite similarly, and better than EBD on average. Though the estimates themselves did not have great reliability for any method, the RSD and SEM methods produced estimates with high coverage rates and power when predicting an outcome with the discrepancy. This suggests that for the purpose of predicting an outcome, either of these methods would be suitable, and neither were greatly impacted by the design factors, indicating that they work well in the full range of research conditions simulated. Researchers studying more complicated models may opt for the SEM discrepancy estimate, while researchers interested in simpler models predicting an outcome with a discrepancy score should find that the easy-to-compute RSD works as well as SEM to predict an outcome. Further research, described in the preceding section, is necessary before concluding whether EBD provides a more accurate and useful estimate than RSD or SEM in other conditions, such as with missing data.

## Data Availability

The raw data supporting the conclusions of this article will be made available by the authors, without undue reservation.

## References

[ref1] Arend M. G. Schäfer T. (2019). Statistical power in two-level models: a tutorial based on Monte Carlo simulation. Psychol. Methods 24, 1–19. doi: 10.1037/met0000195, PMID: 30265048

[ref2] Barooj-Kiakalaee O. Hosseini S. Mohammadpour-Tahmtan R. Hosseini-Tabaghdehi M. Jahanfar S. Esmaeili-Douki Z.. (2022). Paternal postpartum depression’s relationship to maternal pre and postpartum depression, and father-mother dyads marital satisfaction: a structural equation model analysis of a longitudinal study. J. Affect. Disord. 297, 375–380. doi: 10.1016/j.jad.2021.10.110, PMID: 34715195

[ref3] Bar-Sella A. Nof A. Baucom B. R. Goldstein P. Romanov S. Shpakouskaya I.. (2023). The prognostic role of emotion regulation dynamics in the treatment of major depressive disorder. J. Couns. Clin. Psychol. 91, 744–749. doi: 10.1037/ccp0000835, PMID: 37616125

[ref4] Baumann A. A. Kuhlberg J. A. Zayas L. H. (2010). Familism, mother-daughter mutuality, and suicide attempts of adolescent Latinas. J. Fam. Psychol. 24, 616–624. doi: 10.1037/a0020584, PMID: 20954772

[ref5] Blood E. A. Kalish L. A. Shrier L. A. (2013). Estimating heterogeneous intra-class correlation coefficients in dyadic ecological momentary assessment. J. Mod. Appl. Stat. Methods 12, 207–219. doi: 10.22237/jmasm/1367382120

[ref6] Campione-Barr N. Lindell A. K. Giron S. E. (2020). More data, more problems: analytical complications of studying differential family experiences over time: reply to Laird. Dev. Psychol. 56, 978–981. doi: 10.1037/dev0000912, PMID: 32271077

[ref7] Cano A. Johansen A. B. Franz A. (2005). Multilevel analysis of couple congruence on pain, interference, and disability. Pain 118, 369–379. doi: 10.1016/j.pain.2005.09.003, PMID: 16289795 PMC2667887

[ref001] Carr D. Boerner K. (2009). Do spousal discrepancies in marital quality assessments affect psychological adjustment to widowhood? J. Marr. Fam. 71, 495–509. doi: 10.1111/j.1741-3737.2009.00615.x

[ref8] Chen Q. Kwok O. Luo W. Willson V. L. (2010). The impact of ignoring a level of nesting structure in multilevel growth mixture models: a Monte Carlo study. Struct. Equ. Model. 17, 570–589. doi: 10.1080/10705511.2010.510046

[ref9] Chiou J. Spreng R. A. (1996). The reliability of difference scores: a re-examination. J. Consum. Satisf. Dissatisfaction Complain. Behav. 9, 158–167.

[ref10] Clarke P. Wheaton B. (2007). Addressing data sparseness in contextual population research: using cluster analysis to create synthetic neighborhoods. Sociol. Methods Res. 35, 311–351. doi: 10.1177/0049124106292362

[ref11] Cohen J. (1988). Statistical power analysis for the behavioral sciences. 2nd Edn. Mahwah, NJ: Erlbaum.

[ref12] Costigan C. L. Dokis D. P. (2006). Similarities and differences in acculturation among mothers, fathers, and children in immigrant Chinese families. J. Cross-Cult. Psychol. 37, 723–741. doi: 10.1177/0022022106292080

[ref13] Cronbach L. J. Furby L. (1970). How we should measure "change": or should we? Psychol. Bull. 74, 68–80. doi: 10.1037/h0029382

[ref14] Crouter A. C. Davis K. D. Updegraff K. Delgado M. Fortner M. (2006). Mexican American fathers' occupational conditions: links to family members' psychological adjustment. J. Marriage Fam. 68, 843–858. doi: 10.1111/j.1741-3737.2006.00299.x, PMID: 18414596 PMC2293296

[ref15] De Los Reyes A. Kazdin A. E. (2004). Measuring informant discrepancies in clinical child research. Psychol. Assess. 16, 330–334. doi: 10.1037/1040-3590.16.3.330, PMID: 15456389

[ref16] DeVellis R. F. (2006). Classical test theory. Med. Care 44, S50–S59. doi: 10.1097/01.mlr.0000245426.10853.30, PMID: 17060836

[ref002] Gonzalez R. Griffin D. (1997). “On the statistics of interdependence: Treating dyadic data with respect,” in Handbook of personal relationships: Theory, research and interventions (2nd ed.). ed. Duck S. (John Wiley & Sons, Inc.), p. 271–302.

[ref17] Guion K. Mrug S. Windle M. (2009). Predictive value of informant discrepancies in reports of parenting: relations to early adolescents' adjustment. J. Abnorm. Child Psychol. 37, 17–30. doi: 10.1007/s10802-008-9253-5, PMID: 18584134

[ref18] Gulliford M. C. Ukoumunne O. C. Chinn S. (1999). Components of variance and intraclass correlations for the design of community-based surveys and intervention studies: data from the health survey for England 1994. Am. J. Epidemiol. 149, 876–883. doi: 10.1093/oxfordjournals.aje.a009904, PMID: 10221325

[ref19] Kenny D. A. Kashy D. A. Cook W. L. (2006a). “Dyadic indexes” in Dyadic data analysis. eds. Kenny D. Kashy D. Cook W. (New York, NY: Guilford Press), 317–341.

[ref20] Kenny D. A. Kashy D. A. Cook W. L. (2006b). “The measurement of nonindependence” in Dyadic data analysis. eds. Kenny D. Kashy D. Cook W. (New York: Guilford Press), 25–52.

[ref21] Kim S. Y. Chen Q. Li J. Huang X. Moon U. J. (2009). Parent–child acculturation, parenting, and adolescent depressive symptoms in Chinese immigrant families. J. Fam. Psychol. 23, 426–437. doi: 10.1037/a0016019, PMID: 19586205 PMC2746862

[ref22] Kim S. Y. Chen Q. Wang Y. Shen Y. Orozco-Lapray D. (2013). Longitudinal linkages among parent–child acculturation discrepancy, parenting, parent–child sense of alienation, and adolescent adjustment in Chinese immigrant families. Dev. Psychol. 49, 900–912. doi: 10.1037/a0029169, PMID: 22799587 PMC3514557

[ref23] Krull J. L. MacKinnon D. P. (2001). Multilevel modeling of individual and group level mediated effects. Multivar. Behav. Res. 36, 249–277. doi: 10.1207/S15327906MBR3602_06, PMID: 26822111

[ref24] Laird R. D. (2020). Analytical challenges of testing hypotheses of agreement and discrepancy: comment on Campione-Barr, Lindell, and Giron (2020). Dev. Psychol. 56, 970–977. doi: 10.1037/dev0000763, PMID: 32271076

[ref25] Lau A. S. McCabe K. M. Yeh M. Garland A. F. Wood P. A. Hough R. L. (2005). The acculturation gap-distress hypothesis among high-risk Mexican American families. J. Fam. Psychol. 19, 367–375. doi: 10.1037/0893-3200.19.3.367, PMID: 16221017

[ref26] Leidy M. S. Parke R. D. Cladis M. Coltrane S. Duffy S. (2009). Positive marital quality, acculturative stress, and child outcomes among Mexican Americans. J. Marriage Fam. 71, 833–847. doi: 10.1111/j.1741-3737.2009.00638.x

[ref27] Lord F. M. (1963). “Elementary models for measuring change” in Problems in measuring change. ed. Harris C. W. (Madison, WI: University of Wisconsin Press), 21–38.

[ref28] Luo W. Kwok O. (2009). The impacts of ignoring a crossed factor in analyzing cross-classified data. Multivar. Behav. Res. 44, 182–212. doi: 10.1080/00273170902794214, PMID: 26754266

[ref29] Maas C. J. Hox J. J. (2005). Sufficient sample sizes for multilevel modeling. Methodol. Eur. J. Res. Methods Behav. Soc. Sci. 1, 86–92. doi: 10.1027/1614-2241.1.3.86

[ref30] McEnturff A. Chen Q. Turner H. Luo W. (2013) *Evaluating MLM and SEM approaches of estimating acculturation discrepancy: a simulation study.* Paper presented at the 121st annual meeting of the American Psychological Association, Honolulu, Hawaii.

[ref31] McHale S. M. Updegraff K. A. Shanahan L. Crouter A. C. Killoren S. E. (2005). Siblings’ differential treatment in Mexican American families. J. Marriage Fam. 67, 1259–1274. doi: 10.1111/j.1741-3737.2005.00215.x, PMID: 18414595 PMC2293294

[ref32] Moerbeek M. (2004). The consequence of ignoring a level of nesting in multilevel analysis. Multivar. Behav. Res. 39, 129–149. doi: 10.1207/s15327906mbr3901_5, PMID: 26759936

[ref33] Newsom J. T. (2002). A multilevel structural equation model for dyadic data. Struct. Equ. Model. 9, 431–447. doi: 10.1207/S15328007SEM0903_7

[ref34] Raudenbush S. W. (2008). “Many small groups,” in Handbook of multilevel analysis. eds. de Leeuw J. Meijer E. (New York, NY: Springer).

[ref35] Raudenbush S. W. Bryk A. S. (2002). Hierarchical linear models: Applications and data analysis methods. 2nd Edn. Thousand Oaks, CA: Sage Publications, Inc.

[ref36] Russell J. D. Graham R. A. Neill E. L. Weems C. F. (2016). Agreement in youth-parent perceptions of parenting behaviors: a case for testing measurement invariance in reporter discrepancy research. J. Youth Adolesc. 45, 2094–2107. doi: 10.1007/s10964-016-0495-1, PMID: 27289553 PMC7690213

[ref37] Sakaluk J. K. Joel S. Quinn-Nilas C. Camanto O. J. Pevie N. W. Tu E.. (2025). A renewal of dyadic structural equation modeling with latent variables: clarifications, methodological advantages, and new directions. Soc. Personal. Psychol. Compass 19, 1–15. doi: 10.1111/spc3.70045, PMID: 40415499

[ref38] Sayer A. G. Klute M. M. (2005). “Analyzing couples and families: multilevel methods” in Sourcebook of family theory and research. eds. Bengston V. L. Acock A. C. Allen K. R. Dilworth-Anderson P. Klein D. M. (Thousand Oaks, CA: Sage Publications, Inc), 289–313.

[ref39] Schmid B. Allen R. S. Haley P. P. DeCoster J. (2010). Family matters: dyadic agreement in end-of-life medical decision making. The Gerontologist 50, 226–237. doi: 10.1093/geront/gnp166, PMID: 20038541 PMC2838411

[ref40] Stage S. A. (2001). Program evaluation using hierarchical linear modeling with curriculum-based measurement reading probes. Sch. Psychol. Q. 16, 91–112. doi: 10.1521/scpq.16.1.91.19159

[ref41] Stander V. A. Hsiung P. MacDermid S. (2001). The relationship of attributions to marital distress: a comparison of mainland Chinese and U.S. couples. J. Fam. Psychol. 15, 124–134. doi: 10.1037/0893-3200.15.1.124, PMID: 11322080

[ref42] Steele J. S. Ferrer E. Nesselroade J. R. (2013). An idiographic approach to estimating models of dyadic interactions with differential equations. Psychometrika 79, 675–700. doi: 10.1007/s11336-013-9366-9, PMID: 24352513

[ref43] Wang Y. Benner A. D. (2013). Parent-child discrepancies in educational expectations: differential effects of actual versus perceived discrepancies. Child Dev. 85, 891–900. doi: 10.1111/cdev.12171, PMID: 24116710

[ref44] Wheeler L. A. Updegraff K. A. Thayer S. M. (2010). Conflict resolution in Mexican-origin couples: culture, gender, and marital quality. J. Marriage Fam. 72, 991–1005. doi: 10.1111/j.1741-3737.2010.00744.x, PMID: 21278807 PMC3616146

[ref45] Williams R. H. Zimmerman D. W. (1977). The reliability of difference scores when errors are correlated. Educ. Psychol. Meas. 37, 679–689. doi: 10.1177/001316447703700310

[ref46] Zumbo B. D. (1999). “The simple difference score as an inherently poor measure of change: some reality, much mythology” in Advances in social science methodology, vol 5. ed. Thompson B. (Leeds, England: Emerald Group Publishing Limited), 269–304.

