# Supplementary material for: Comparing raw score difference, multilevel modeling, and structural equation modeling methods for estimating discrepancy in dyads
Source: Front Psychol. 2025 Jun 19;16:1499076. doi: 10.3389/fpsyg.2025.1499076 (PMC12225616; doi:10.3389/fpsyg.2025.1499076)
Supplement: Supplementary file 1 [file Supplementary_file_1.docx]

**Appendix 1-A Data Generation using SAS**

options symbolgen;

libname dyad 'C:/siml';

PROC IMPORT OUT=dyad.macro DATAFILE= "C:\macro.xlsx"

DBMS=xlsx REPLACE;

SHEET="Sheet1";

GETNAMES=YES;

RUN;

%macro doit;

%let id=%sysfunc(open(dyad.macro));

%let NObs=%sysfunc(attrn(&id,NOBS));

%syscall set(id);

%do i=1 %to 216;

%let rc=%sysfunc(fetchobs(&id,&i));

proc printto log="c:\siml\log_&simid"; run;

proc printto print="c:\siml\output_&simid";run;

%macro iterate;

%do k=1 %to &NumSamples;

*///////////////////////////////////////////////////////////////////////////////////////////////////////////////////////////////////////////////;

*Between and within with means of 0, true score variance = 1, effect size with variance added later randomly to one dyad member.

*///////////////////////////////////////////////////////////////////////////////////////////////////////////////////////////////////////////////;

*BETWEEN variance;

data between;

do i = 1 to &N;

between = sqrt(&bvar)*rannor(&k);

output;

end;

RUN;

*WITHIN variance;

data within;

do m = 1 to &N;

within1 = sqrt(&wvar)*rannor(&k);

within2 = sqrt(&wvar)*rannor(&k);

output;

end;

RUN;

*ERROR with variance;

data error;

do j = 1 to &N;

error1 = .5*rannor(&k);

error2 = .5*rannor(&k);

output;

end;

run;

*/////////////////////////////////////////////////////////////////////////////////////;

*Add in Effect Size;

*/////////////////////////////////////////////////////////////////////////////////////;

data es;

do i = 1 to &N;

es=rand('Normal', &es, &esvar);

output;

end;

run;

proc surveyselect data=es out=split samprate=.5 outall noprint;

run;

data es;

set split;

if selected=1 then es1=es;

if selected=1 then es2=0;

if selected=0 then es1=0;

if selected=0 then es2=es;

run;

data work.dyad&k (drop=i m j);

do dyadid=1 to &N;

merge between within es error;

iterid = &k;

simid = &simid;

true1=between+within1+es1;

true2=between+within2+es2;

raw1=true1+error1;

raw2=true2+error2;

difftrue=true2-true1;

rsd=raw2-raw1;

output;

end;

run;

quit;

%end;

%mend iterate;

quit;

%iterate;

%macro append;

%do z=1 %to &NumSamples;

proc append base=dyad.simid&simid data=work.dyad&z;

run;

%end;

%mend append;

%append;

proc sort data=dyad.simid&simid;

by iterid;

run;

proc means data=dyad.simid&simid mean var noprint;

by iterid;

var between within1 within2 es error1 error2 true1 true2 raw1 raw2;

output out=dyad.qc&simid(drop=_TYPE_ _FREQ_) mean=between_mean within1_mean within2_mean es_mean error1_mean error2_mean true1_mean true2_mean raw1_mean raw2_mean

var=between_var within1_var within2_var es_var error1_var error2_var true1_var true2_var raw1_var raw2_var;

run;

data dyad.qc&simid;

set dyad.qc&simid;

simid=&simid;

run;

end;

let id=sysfunc(close(&id));

mend doit;

doit;

**Appendix 1-B Mplus SEM Discrepancy Score Generation**

TITLE: MPLUS SEM dyadic discrepancy

DATA: FILE = sim3.dat;

TYPE = montecarlo;

VARIABLE: NAMES are true1 true2 raw1 raw2 outcomed outcomei;

ANALYSIS: TYPE = RANDOM;

ALGORITH = INTEGRATION;

MODEL: int BY raw1 raw2;

slpdiff BY raw1*-.5 raw2*.5;

slpdiff ON outcomei;

SAVEDATA: FILE IS output3.csv;

SAVE IS FSCORES;

**Appendix 1-C Data Analysis**

libname dyad 'D:/siml';

*Read macro sheet which has simid and numsamples, and the macro doit references this to know how many simids to do;

PROC IMPORT OUT=dyad.macro DATAFILE= "D:\macro.xlsx"

DBMS=xlsx REPLACE;

SHEET="Sheet1" ;

GETNAMES=YES;

RUN;

%macro doit;

%let id=%sysfunc(open(dyad.macro));

%let NObs=%sysfunc(attrn(&id,NOBS));

%syscall set(id);

%do i=179 %to 179;

%let rc=%sysfunc(fetchobs(&id,&i));

%put # # # Processing Name # # #;

proc printto log="c:\siml719\logsem_&simid"; run;

proc printto print="c:\siml719\outputsem_&simid";run;

%let sid=&simid;

/*%let sid=1;*/

data dyad.outb&sid;

set dyad.outb&sid;

rename sem=sem_old;

run;

data dyad.outb&sid;

set dyad.outb&sid;

rename sem_mp=sem;

run;

proc means data=dyad.outb&sid noprint mean;

by iterid;

var sem;

output out=sem&sid(drop= _FREQ_ _TYPE_) mean=semmean;

run;

data dyad.outb&sid;

set dyad.outb&sid;

semabs=abs(sem);

rbsem=(sem-difftrue)/difftrue;

run;

data dyad.iter&sid;

merge dyad.iter&sid sem&sid;

by iterid;

run;

*Compute reliabilities of estimates;

proc means data=dyad.outb&sid noprint mean var std;

by iterid;

var difftrue raw1 raw2 rsd diz res ebd sem;

output out=reliability mean=difftruemean dizmean resmean ebdmean semmean var=vardifftrue varrsd vardiz varres varebd varsem;

run;

*this creates an iteration level reliability estimate. will merge into another iteration level file, NOT just fill it in down the column;

data reliability;

set reliability;

relrsd=vardifftrue/varrsd;

reldiz=vardifftrue/vardiz;

relres=vardifftrue/varres;

relebd=vardifftrue/varebd;

relsem=vardifftrue/varsem;

run;

data dyad.iter&sid;

merge dyad.iter&sid reliability;

by iterid;

run;

********************************************;

*14.GENERATE OUTCOME USING REGRESSION MODEL*;

********************************************;

*this needs to have a model for dist and indistinguishable.;

*The distinguishable model is shown already with the group variable;

*the indistinguishable dyad shouldnt have group or the interaction;

data dyad.outb&sid;

set dyad.outb&sid;

by iterid;

outcome_d=0.5+0.8*difftrueabs+.2*group+.1*difftrueabs*group+rannor(0);

outcome_i=0.5+0.8*difftrueabs+rannor(0);

difftrueabsg=difftrueabs*group;

rsdabsg=rsdabs*group;

dizabsg=dizabs*group;

resabsg=resabs*group;

ebdabsg=ebdabs*group;

semabsg=semabs*group;

run;

/*compare parameter estimates in the regression models using different discrepancy scores - evaluates accuracy of prediction*/

/*options nonotes;*/

ods trace on /listing;

ods select none;

ods graphics off;

ods exclude all;

ods noresults;

proc reg data=dyad.outb&sid;

by iterid;

model outcome_d= difftrueabs group difftrueabsg/stb clb;

model outcome_d=rsdabs group rsdabsg/stb clb;

model outcome_d=ebdabs group ebdabsg/stb clb;

model outcome_d=dizabs group dizabsg/stb clb;

model outcome_d=resabs group resabsg/stb clb;

model outcome_d=semabs group semabsg/stb clb;

model outcome_i= difftrueabs /stb clb;

model outcome_i=rsdabs /stb clb;

model outcome_i=ebdabs /stb clb;

model outcome_i=dizabs /stb clb;

model outcome_i=resabs /stb clb;

model outcome_i=semabs /stb clb;

ods output ANOVA=anova;

ods output ParameterEstimates=parmest;

ods output FitStatistics =fitstat;

run;

ods output close;

ods trace off;

ods graphics on;

ods exclude none;

ods results;

ods select all;

options notes;

********************************************;

*16.TRANPOSE ANOVA UNIVARIATE TO MULTIVAR *;

********************************************;

data ANOVA2;

set ANOVA;

by iterid;

if Source = 'Error' then delete;

if Source = 'Corrected Total' then delete;

if Model = 'MODEL1' then iv = 'difftrue_d';

if Model = 'MODEL2' then iv = 'rsd_d';

if Model = 'MODEL3' then iv = 'ebd_d';

if Model = 'MODEL4' then iv = 'diz_d';

if Model = 'MODEL5' then iv = 'res_d';

if Model = 'MODEL6' then iv = 'sem_d';

if Model = 'MODEL7' then iv = 'difftrue_i';

if Model = 'MODEL8' then iv = 'rsd_i';

if Model = 'MODEL9' then iv = 'ebd_i';

if Model = 'MODEL10' then iv = 'diz_i';

if Model = 'MODEL11' then iv = 'res_i';

if Model = 'MODEL12' then iv = 'sem_i';

z = 1;

drop Model Dependent Source DF SS MS;

run;

proc transpose data = ANOVA2 out = ANOVA3 prefix = F_;

by iterid z;

id iv;

var FValue;

run;

proc transpose data = ANOVA2 out = ANOVA4 prefix = p_;

by iterid z;

id iv;

var ProbF;

run;

data ANOVA5;

merge ANOVA3 ANOVA4;

run;

/*to compute power later based on overall significance of model from ANOVA results*/

data ANOVA6(drop=_NAME_);

set ANOVA5;

if p_difftrue_d lt .05 then p_difftrue_count_d = 1; else p_difftrue_count_d = 0;

if p_rsd_d lt .05 then p_rsd_count_d = 1; else p_rsd_count_d = 0;

if p_ebd_d lt .05 then p_ebd_count_d = 1; else p_ebd_count_d = 0;

if p_diz_d lt .05 then p_diz_count_d = 1; else p_diz_count_d = 0;

if p_res_d lt .05 then p_res_count_d = 1; else p_res_count_d = 0;

if p_sem_d lt .05 then p_sem_count_d = 1; else p_sem_count_d = 0;

if p_difftrue_i lt .05 then p_difftrue_count_i = 1; else p_difftrue_count_i = 0;

if p_rsd_i lt .05 then p_rsd_count_i = 1; else p_rsd_count_i = 0;

if p_ebd_i lt .05 then p_ebd_count_i = 1; else p_ebd_count_i = 0;

if p_diz_i lt .05 then p_diz_count_i = 1; else p_diz_count_i = 0;

if p_res_i lt .05 then p_res_count_i = 1; else p_res_count_i = 0;

if p_sem_i lt .05 then p_sem_count_i = 1; else p_sem_count_i = 0;

run;

proc datasets lib=work memtype=data noprint;

modify parmest;

attrib _all_ label=' ';

run;

********************************************;

*18.REARRANGING PARMEST FOR DTRUE *;

********************************************;

data dtruetemp1;

set parmest (rename=(df=int_df_dtrue_d

estimate = int_est_dtrue_d

StdErr=int_stderr_dtrue_d

tvalue=int_tvalue_dtrue_d

probt=int_pvalue_dtrue_d

LowerCL=int_lcl_dtrue_d

UpperCL=int_ucl_dtrue_d

StandardizedEst = int_stdest_dtrue_d));

if Model = 'MODEL1' and Variable = 'Intercept';

iv = 'difftrue_d';

run;

data dtruetemp2;

set parmest(rename=(df=slopediff_df_dtrue_d

estimate = slopediff_dtrue_d

StdErr=slopediff_stderr_dtrue_d

tvalue=slopediff_tvalue_dtrue_d

probt=slopediff_pvalue_dtrue_d

LowerCL=slopediff_lcl_dtrue_d

UpperCL=slopediff_ucl_dtrue_d

StandardizedEst = slopediff_stdest_dtrue_d));

if Model = 'MODEL1' and Variable = 'difftrueabs';

iv = 'difftrue_d';

run;

data dtruetemp3;

set parmest(rename=(df=slopeg_df_dtrue_d

estimate = slopeg_dtrue_d

StdErr=slopeg_stderr_dtrue_d

tvalue=slopeg_tvalue_dtrue_d

probt=slopeg_pvalue_dtrue_d

LowerCL=slopeg_lcl_dtrue_d

UpperCL=slopeg_ucl_dtrue_d

StandardizedEst = slopeg_stdest_dtrue_d));

if Model = 'MODEL1' and Variable = 'group';

iv = 'difftrue_d';

run;

data dtruetemp4;

set parmest(rename=(df=slopeint_df_dtrue_d

estimate = slopeint_dtrue_d

StdErr=slopeint_stderr_dtrue_d

tvalue=slopeint_tvalue_dtrue_d

probt=slopeint_pvalue_dtrue_d

LowerCL=slopeint_lcl_dtrue_d

UpperCL=slopeint_ucl_dtrue_d

StandardizedEst = slopeint_stdest_dtrue_d));

if Model = 'MODEL1' and Variable = 'difftrueabsg';

iv = 'difftrue_d';

run;

data dtrue_d;

merge dtruetemp1 dtruetemp2 dtruetemp3 dtruetemp4;

run;

proc datasets lib=work memtype=data noprint;

modify dtrue_d;

attrib _all_ label=' ';

run;

********************************************;

*18.REARRANGING PARMEST FOR RSD *;

********************************************;

data rsdtemp1;

set parmest (rename=(df=int_df_rsd_d

estimate = int_est_rsd_d

StdErr=int_stderr_rsd_d

tvalue=int_tvalue_rsd_d

probt=int_pvalue_rsd_d

LowerCL=int_lcl_rsd_d

UpperCL=int_ucl_rsd_d

StandardizedEst = int_stdest_rsd_d));

if Model = 'MODEL2' and Variable = 'Intercept';

iv = 'rsd_d';

run;

data rsdtemp2;

set parmest(rename=(df=slopediff_df_rsd_d

estimate = slopediff_rsd_d

StdErr=slopediff_stderr_rsd_d

tvalue=slopediff_tvalue_rsd_d

probt=slopediff_pvalue_rsd_d

LowerCL=slopediff_lcl_rsd_d

UpperCL=slopediff_ucl_rsd_d

StandardizedEst = slopediff_stdest_rsd_d));

if Model = 'MODEL2' and Variable = 'rsdabs';

iv = 'rsd_d';

run;

data rsdtemp3;

set parmest(rename=(df=slopeg_df_rsd_d

estimate = slopeg_rsd_d

StdErr=slopeg_stderr_rsd_d

tvalue=slopeg_tvalue_rsd_d

probt=slopeg_pvalue_rsd_d

LowerCL=slopeg_lcl_rsd_d

UpperCL=slopeg_ucl_rsd_d

StandardizedEst = slopeg_stdest_rsd_d));

if Model = 'MODEL2' and Variable = 'group';

iv = 'rsd_d';

run;

data rsdtemp4;

set parmest(rename=(df=slopeint_df_rsd_d

estimate = slopeint_rsd_d

StdErr=slopeint_stderr_rsd_d

tvalue=slopeint_tvalue_rsd_d

probt=slopeint_pvalue_rsd_d

LowerCL=slopeint_lcl_rsd_d

UpperCL=slopeint_ucl_rsd_d

StandardizedEst = slopeint_stdest_rsd_d));

if Model = 'MODEL2' and Variable = 'rsdabsg';

iv = 'rsd_d';

run;

data rsd_d;

merge rsdtemp1 rsdtemp2 rsdtemp3 rsdtemp4;

run;

proc datasets lib=work memtype=data noprint;

modify rsd_d;

attrib _all_ label=' ';

run;

********************************************;

*18.REARRANGING PARMEST FOR EBD *;

********************************************;

data ebdtemp1;

set parmest (rename=(df=int_df_ebd_d

estimate = int_est_ebd_d

StdErr=int_stderr_ebd_d

tvalue=int_tvalue_ebd_d

probt=int_pvalue_ebd_d

LowerCL=int_lcl_ebd_d

UpperCL=int_ucl_ebd_d

StandardizedEst = int_stdest_ebd_d));

if Model = 'MODEL3' and Variable = 'Intercept';

iv = 'ebd_d';

run;

data ebdtemp2;

set parmest(rename=(df=slopediff_df_ebd_d

estimate = slopediff_ebd_d

StdErr=slopediff_stderr_ebd_d

tvalue=slopediff_tvalue_ebd_d

probt=slopediff_pvalue_ebd_d

LowerCL=slopediff_lcl_ebd_d

UpperCL=slopediff_ucl_ebd_d

StandardizedEst = slopediff_stdest_ebd_d));

if Model = 'MODEL3' and Variable = 'ebdabs';

iv = 'ebd_d';

run;

data ebdtemp3;

set parmest(rename=(df=slopeg_df_ebd_d

estimate = slopeg_ebd_d

StdErr=slopeg_stderr_ebd_d

tvalue=slopeg_tvalue_ebd_d

probt=slopeg_pvalue_ebd_d

LowerCL=slopeg_lcl_ebd_d

UpperCL=slopeg_ucl_ebd_d

StandardizedEst = slopeg_stdest_ebd_d));

if Model = 'MODEL3' and Variable = 'group';

iv = 'ebd_d';

run;

data ebdtemp4;

set parmest(rename=(df=slopeint_df_ebd_d

estimate = slopeint_ebd_d

StdErr=slopeint_stderr_ebd_d

tvalue=slopeint_tvalue_ebd_d

probt=slopeint_pvalue_ebd_d

LowerCL=slopeint_lcl_ebd_d

UpperCL=slopeint_ucl_ebd_d

StandardizedEst = slopeint_stdest_ebd_d));

if Model = 'MODEL3' and Variable = 'ebdabsg';

iv = 'ebd_d';

run;

data ebd_d;

merge ebdtemp1 ebdtemp2 ebdtemp3 ebdtemp4;

run;

proc datasets lib=work memtype=data noprint;

modify ebd_d;

attrib _all_ label=' ';

run;

********************************************;

*18.REARRANGING PARMEST FOR DIZ *;

********************************************;

data diztemp1;

set parmest (rename=(df=int_df_diz_d

estimate = int_est_diz_d

StdErr=int_stderr_diz_d

tvalue=int_tvalue_diz_d

probt=int_pvalue_diz_d

LowerCL=int_lcl_diz_d

UpperCL=int_ucl_diz_d

StandardizedEst = int_stdest_diz_d));

if Model = 'MODEL4' and Variable = 'Intercept';

iv = 'diz_d';

run;

data diztemp2;

set parmest(rename=(df=slopediff_df_diz_d

estimate = slopediff_diz_d

StdErr=slopediff_stderr_diz_d

tvalue=slopediff_tvalue_diz_d

probt=slopediff_pvalue_diz_d

LowerCL=slopediff_lcl_diz_d

UpperCL=slopediff_ucl_diz_d

StandardizedEst = slopediff_stdest_diz_d));

if Model = 'MODEL4' and Variable = 'dizabs';

iv = 'diz_d';

run;

data diztemp3;

set parmest(rename=(df=slopeg_df_diz_d

estimate = slopeg_diz_d

StdErr=slopeg_stderr_diz_d

tvalue=slopeg_tvalue_diz_d

probt=slopeg_pvalue_diz_d

LowerCL=slopeg_lcl_diz_d

UpperCL=slopeg_ucl_diz_d

StandardizedEst = slopeg_stdest_diz_d));

if Model = 'MODEL4' and Variable = 'group';

iv = 'diz_d';

run;

data diztemp4;

set parmest(rename=(df=slopeint_df_diz_d

estimate = slopeint_diz_d

StdErr=slopeint_stderr_diz_d

tvalue=slopeint_tvalue_diz_d

probt=slopeint_pvalue_diz_d

LowerCL=slopeint_lcl_diz_d

UpperCL=slopeint_ucl_diz_d

StandardizedEst = slopeint_stdest_diz_d));

if Model = 'MODEL4' and Variable = 'dizabsg';

iv = 'diz_d';

run;

data diz_d;

merge diztemp1 diztemp2 diztemp3 diztemp4;

run;

proc datasets lib=work memtype=data noprint;

modify diz_d;

attrib _all_ label=' ';

run;

********************************************;

*18.REARRANGING PARMEST FOR RES *;

********************************************;

data restemp1;

set parmest (rename=(df=int_df_res_d

estimate = int_est_res_d

StdErr=int_stderr_res_d

tvalue=int_tvalue_res_d

probt=int_pvalue_res_d

LowerCL=int_lcl_res_d

UpperCL=int_ucl_res_d

StandardizedEst = int_stdest_res_d));

if Model = 'MODEL5' and Variable = 'Intercept';

iv = 'res_d';

run;

data restemp2;

set parmest(rename=(df=slopediff_df_res_d

estimate = slopediff_res_d

StdErr=slopediff_stderr_res_d

tvalue=slopediff_tvalue_res_d

probt=slopediff_pvalue_res_d

LowerCL=slopediff_lcl_res_d

UpperCL=slopediff_ucl_res_d

StandardizedEst = slopediff_stdest_res_d));

if Model = 'MODEL5' and Variable = 'resabs';

iv = 'res_d';

run;

data restemp3;

set parmest(rename=(df=slopeg_df_res_d

estimate = slopeg_res_d

StdErr=slopeg_stderr_res_d

tvalue=slopeg_tvalue_res_d

probt=slopeg_pvalue_res_d

LowerCL=slopeg_lcl_res_d

UpperCL=slopeg_ucl_res_d

StandardizedEst = slopeg_stdest_res_d));

if Model = 'MODEL5' and Variable = 'group';

iv = 'res_d';

run;

data restemp4;

set parmest(rename=(df=slopeint_df_res_d

estimate = slopeint_res_d

StdErr=slopeint_stderr_res_d

tvalue=slopeint_tvalue_res_d

probt=slopeint_pvalue_res_d

LowerCL=slopeint_lcl_res_d

UpperCL=slopeint_ucl_res_d

StandardizedEst = slopeint_stdest_res_d));

if Model = 'MODEL5' and Variable = 'resabsg';

iv = 'res_d';

run;

data res_d;

merge restemp1 restemp2 restemp3 restemp4;

run;

proc datasets lib=work memtype=data noprint;

modify res_d;

attrib _all_ label=' ';

run;

********************************************;

*18.REARRANGING PARMEST FOR SEM *;

********************************************;

data semtemp1;

set parmest (rename=(df=int_df_sem_d

estimate = int_est_sem_d

StdErr=int_stderr_sem_d

tvalue=int_tvalue_sem_d

probt=int_pvalue_sem_d

LowerCL=int_lcl_sem_d

UpperCL=int_ucl_sem_d

StandardizedEst = int_stdest_sem_d));

if Model = 'MODEL6' and Variable = 'Intercept';

iv = 'sem_d';

run;

data semtemp2;

set parmest(rename=(df=slopediff_df_sem_d

estimate = slopediff_sem_d

StdErr=slopediff_stderr_sem_d

tvalue=slopediff_tvalue_sem_d

probt=slopediff_pvalue_sem_d

LowerCL=slopediff_lcl_sem_d

UpperCL=slopediff_ucl_sem_d

StandardizedEst = slopediff_stdest_sem_d));

if Model = 'MODEL6' and Variable = 'semabs';

iv = 'sem_d';

run;

data semtemp3;

set parmest(rename=(df=slopeg_df_sem_d

estimate = slopeg_sem_d

StdErr=slopeg_stderr_sem_d

tvalue=slopeg_tvalue_sem_d

probt=slopeg_pvalue_sem_d

LowerCL=slopeg_lcl_sem_d

UpperCL=slopeg_ucl_sem_d

StandardizedEst = slopeg_stdest_sem_d));

if Model = 'MODEL6' and Variable = 'group';

iv = 'sem_d';

run;

data semtemp4;

set parmest(rename=(df=slopeint_df_sem_d

estimate = slopeint_sem_d

StdErr=slopeint_stderr_sem_d

tvalue=slopeint_tvalue_sem_d

probt=slopeint_pvalue_sem_d

LowerCL=slopeint_lcl_sem_d

UpperCL=slopeint_ucl_sem_d

StandardizedEst = slopeint_stdest_sem_d));

if Model = 'MODEL6' and Variable = 'semabsg';

iv = 'sem_d';

run;

data sem_d;

merge semtemp1 semtemp2 semtemp3 semtemp4;

run;

proc datasets lib=work memtype=data noprint;

modify sem_d;

attrib _all_ label=' ';

run;

********************************************;

*18.REARRANGING PARMEST FOR DTRUE *;

********************************************;

data dtruetemp1;

set parmest (rename=(df=int_df_dtrue_i

estimate = int_est_dtrue_i

StdErr=int_stderr_dtrue_i

tvalue=int_tvalue_dtrue_i

probt=int_pvalue_dtrue_i

LowerCL=int_lcl_dtrue_i

UpperCL=int_ucl_dtrue_i

StandardizedEst = int_stdest_dtrue_i));

if Model = 'MODEL7' and Variable = 'Intercept';

iv = 'difftrue_i';

run;

data dtruetemp2;

set parmest(rename=(df=slopediff_df_dtrue_i

estimate = slopediff_dtrue_i

StdErr=slopediff_stderr_dtrue_i

tvalue=slopediff_tvalue_dtrue_i

probt=slopediff_pvalue_dtrue_i

LowerCL=slopediff_lcl_dtrue_i

UpperCL=slopediff_ucl_dtrue_i

StandardizedEst = slopediff_stdest_dtrue_i));

if Model = 'MODEL1' and Variable = 'difftrueabs';

iv = 'difftrue_i';

run;

data dtrue_i;

merge dtruetemp1 dtruetemp2;

run;

proc datasets lib=work memtype=data noprint;

modify dtrue_i;

attrib _all_ label=' ';

run;

********************************************;

*18.REARRANGING PARMEST FOR RSD *;

********************************************;

data rsdtemp1;

set parmest (rename=(df=int_df_rsd_i

estimate = int_est_rsd_i

StdErr=int_stderr_rsd_i

tvalue=int_tvalue_rsd_i

probt=int_pvalue_rsd_i

LowerCL=int_lcl_rsd_i

UpperCL=int_ucl_rsd_i

StandardizedEst = int_stdest_rsd_i));

if Model = 'MODEL2' and Variable = 'Intercept';

iv = 'rsd_i';

run;

data rsdtemp2;

set parmest(rename=(df=slopediff_df_rsd_i

estimate = slopediff_rsd_i

StdErr=slopediff_stderr_rsd_i

tvalue=slopediff_tvalue_rsd_i

probt=slopediff_pvalue_rsd_i

LowerCL=slopediff_lcl_rsd_i

UpperCL=slopediff_ucl_rsd_i

StandardizedEst = slopediff_stdest_rsd_i));

if Model = 'MODEL2' and Variable = 'rsdabs';

iv = 'rsd_i';

run;

data rsd_i;

merge rsdtemp1 rsdtemp2;

run;

proc datasets lib=work memtype=data noprint;

modify rsd_i;

attrib _all_ label=' ';

run;

********************************************;

*18.REARRANGING PARMEST FOR EBD *;

********************************************;

data ebdtemp1;

set parmest (rename=(df=int_df_ebd_i

estimate = int_est_ebd_i

StdErr=int_stderr_ebd_i

tvalue=int_tvalue_ebd_i

probt=int_pvalue_ebd_i

LowerCL=int_lcl_ebd_i

UpperCL=int_ucl_ebd_i

StandardizedEst = int_stdest_ebd_i));

if Model = 'MODEL3' and Variable = 'Intercept';

iv = 'ebd_i';

run;

data ebdtemp2;

set parmest(rename=(df=slopediff_df_ebd_i

estimate = slopediff_ebd_i

StdErr=slopediff_stderr_ebd_i

tvalue=slopediff_tvalue_ebd_i

probt=slopediff_pvalue_ebd_i

LowerCL=slopediff_lcl_ebd_i

UpperCL=slopediff_ucl_ebd_i

StandardizedEst = slopediff_stdest_ebd_i));

if Model = 'MODEL3' and Variable = 'ebdabs';

iv = 'ebd_i';

run;

data ebd_i;

merge ebdtemp1 ebdtemp2;

run;

proc datasets lib=work memtype=data noprint;

modify ebd_i;

attrib _all_ label=' ';

run;

********************************************;

*18.REARRANGING PARMEST FOR DIZ *;

********************************************;

data diztemp1;

set parmest (rename=(df=int_df_diz_i

estimate = int_est_diz_i

StdErr=int_stderr_diz_i

tvalue=int_tvalue_diz_i

probt=int_pvalue_diz_i

LowerCL=int_lcl_diz_i

UpperCL=int_ucl_diz_i

StandardizedEst = int_stdest_diz_i));

if Model = 'MODEL4' and Variable = 'Intercept';

iv = 'diz_i';

run;

data diztemp2;

set parmest(rename=(df=slopediff_df_diz_i

estimate = slopediff_diz_i

StdErr=slopediff_stderr_diz_i

tvalue=slopediff_tvalue_diz_i

probt=slopediff_pvalue_diz_i

LowerCL=slopediff_lcl_diz_i

UpperCL=slopediff_ucl_diz_i

StandardizedEst = slopediff_stdest_diz_i));

if Model = 'MODEL4' and Variable = 'dizabs';

iv = 'diz_i';

run;

data diz_i;

merge diztemp1 diztemp2;

run;

proc datasets lib=work memtype=data noprint;

modify diz_i;

attrib _all_ label=' ';

run;

********************************************;

*18.REARRANGING PARMEST FOR RES *;

********************************************;

data restemp1;

set parmest (rename=(df=int_df_res_i

estimate = int_est_res_i

StdErr=int_stderr_res_i

tvalue=int_tvalue_res_i

probt=int_pvalue_res_i

LowerCL=int_lcl_res_i

UpperCL=int_ucl_res_i

StandardizedEst = int_stdest_res_i));

if Model = 'MODEL5' and Variable = 'Intercept';

iv = 'res_i';

run;

data restemp2;

set parmest(rename=(df=slopediff_df_res_i

estimate = slopediff_res_i

StdErr=slopediff_stderr_res_i

tvalue=slopediff_tvalue_res_i

probt=slopediff_pvalue_res_i

LowerCL=slopediff_lcl_res_i

UpperCL=slopediff_ucl_res_i

StandardizedEst = slopediff_stdest_res_i));

if Model = 'MODEL5' and Variable = 'resabs';

iv = 'res_i';

run;

data res_i;

merge restemp1 restemp2;

run;

proc datasets lib=work memtype=data noprint;

modify res_i;

attrib _all_ label=' ';

run;

********************************************;

*18.REARRANGING PARMEST FOR SEM *;

********************************************;

data semtemp1;

set parmest (rename=(df=int_df_sem_i

estimate = int_est_sem_i

StdErr=int_stderr_sem_i

tvalue=int_tvalue_sem_i

probt=int_pvalue_sem_i

LowerCL=int_lcl_sem_i

UpperCL=int_ucl_sem_i

StandardizedEst = int_stdest_sem_i));

if Model = 'MODEL6' and Variable = 'Intercept';

iv = 'sem_i';

run;

data semtemp2;

set parmest(rename=(df=slopediff_df_sem_i

estimate = slopediff_sem_i

StdErr=slopediff_stderr_sem_i

tvalue=slopediff_tvalue_sem_i

probt=slopediff_pvalue_sem_i

LowerCL=slopediff_lcl_sem_i

UpperCL=slopediff_ucl_sem_i

StandardizedEst = slopediff_stdest_sem_i));

if Model = 'MODEL6' and Variable = 'semabs';

iv = 'sem_i';

run;

data sem_i;

merge semtemp1 semtemp2;

run;

proc datasets lib=work memtype=data noprint;

modify sem_i;

attrib _all_ label=' ';

run;

data regparms;

merge dtrue_d rsd_d ebd_d diz_d res_d sem_d dtrue_i rsd_i ebd_i diz_i res_i sem_i;

by iterid;

simid=&simid;

rb_int_est_dtrue_d=(int_est_dtrue_d-.5)/.5;

rb_slopediff_dtrue_d=(slopediff_dtrue_d-.8)/.8;

rb_slopeg_dtrue_d=(slopeg_dtrue_d-.2)/.2;

rb_slopeint_dtrue_d=(slopeint_dtrue_d-.1)/.1;

rb_int_est_rsd_d=(int_est_rsd_d-.5)/.5;

rb_slopediff_rsd_d=(slopediff_rsd_d-.8)/.8;

rb_slopeg_rsd_d=(slopeg_rsd_d-.2)/.2;

rb_slopeint_rsd_d=(slopeint_rsd_d-.1)/.1;

rb_int_est_ebd_d=(int_est_ebd_d-.5)/.5;

rb_slopediff_ebd_d=(slopediff_ebd_d-.8)/.8;

rb_slopeg_ebd_d=(slopeg_ebd_d-.2)/.2;

rb_slopeint_ebd_d=(slopeint_ebd_d-.1)/.1;

rb_int_est_diz_d=(int_est_diz_d-.5)/.5;

rb_slopediff_diz_d=(slopediff_diz_d-.8)/.8;

rb_slopeg_diz_d=(slopeg_diz_d-.2)/.2;

rb_slopeint_diz_d=(slopeint_diz_d-.1)/.1;

rb_int_est_res_d=(int_est_res_d-.5)/.5;

rb_slopediff_res_d=(slopediff_res_d-.8)/.8;

rb_slopeg_res_d=(slopeg_res_d-.2)/.2;

rb_slopeint_res_d=(slopeint_res_d-.1)/.1;

rb_int_est_sem_d=(int_est_sem_d-.5)/.5;

rb_slopediff_sem_d=(slopediff_sem_d-.8)/.8;

rb_slopeg_sem_d=(slopeg_sem_d-.2)/.2;

rb_slopeint_sem_d=(slopeint_sem_d-.1)/.1;

rb_int_est_dtrue_i=(int_est_dtrue_i-.5)/.5;

rb_slopediff_dtrue_i=(slopediff_dtrue_i-.8)/.8;

rb_int_est_rsd_i=(int_est_rsd_i-.5)/.5;

rb_slopediff_rsd_i=(slopediff_rsd_i-.8)/.8;

rb_int_est_ebd_i=(int_est_ebd_i-.5)/.5;

rb_slopediff_ebd_i=(slopediff_ebd_i-.8)/.8;

rb_int_est_diz_i=(int_est_diz_i-.5)/.5;

rb_slopediff_diz_i=(slopediff_diz_i-.8)/.8;

rb_int_est_res_i=(int_est_res_i-.5)/.5;

rb_slopediff_res_i=(slopediff_res_i-.8)/.8;

rb_int_est_sem_i=(int_est_sem_i-.5)/.5;

rb_slopediff_sem_i=(slopediff_sem_i-.8)/.8;

run;

proc means data=regparms std noprint;

var int_est_dtrue_d

slopediff_dtrue_d

slopeg_dtrue_d

slopeint_dtrue_d

int_est_rsd_d

slopediff_rsd_d

slopeg_rsd_d

slopeint_rsd_d

int_est_ebd_d

slopediff_ebd_d

slopeg_ebd_d

slopeint_ebd_d

int_est_diz_d

slopediff_diz_d

slopeg_diz_d

slopeint_diz_d

int_est_res_d

slopediff_res_d

slopeg_res_d

slopeint_res_d

int_est_sem_d

slopediff_sem_d

slopeg_sem_d

slopeint_sem_d

int_est_dtrue_i

slopediff_dtrue_i

int_est_rsd_i

slopediff_rsd_i

int_est_ebd_i

slopediff_ebd_i

int_est_diz_i

slopediff_diz_i

int_est_res_i

slopediff_res_i

int_est_sem_i

slopediff_sem_i;

output out=regstd std=int_est_dtrue_d_std

slopediff_dtrue_d_std

slopeg_dtrue_d_std

slopeint_dtrue_d_std

int_est_rsd_d_std

slopediff_rsd_d_std

slopeg_rsd_d_std

slopeint_rsd_d_std

int_est_ebd_d_std

slopediff_ebd_d_std

slopeg_ebd_d_std

slopeint_ebd_d_std

int_est_diz_d_std

slopediff_diz_d_std

slopeg_diz_d_std

slopeint_diz_d_std

int_est_res_d_std

slopediff_res_d_std

slopeg_res_d_std

slopeint_res_d_std

int_est_sem_d_std

slopediff_sem_d_std

slopeg_sem_d_std

slopeint_sem_d_std

int_est_dtrue_i_std

slopediff_dtrue_i_std

int_est_rsd_i_std

slopediff_rsd_i_std

int_est_ebd_i_std

slopediff_ebd_i_std

int_est_diz_i_std

slopediff_diz_i_std

int_est_res_i_std

slopediff_res_i_std

int_est_sem_i_std

slopediff_sem_i_std;

run;

data regstd;

set regstd;

simid=&simid;

run;

data regparms;

merge regparms regstd;

by simid;

rb_int_stderr_dtrue_d=(int_stderr_dtrue_d-int_est_dtrue_d_std)/int_est_dtrue_d_std;

rb_slopediff_stderr_dtrue_d=(slopediff_stderr_dtrue_d-slopediff_dtrue_d_std)/slopediff_dtrue_d_std;

rb_slopeg_stderr_dtrue_d =(slopeg_stderr_dtrue_d -slopeg_dtrue_d_std)/slopeg_dtrue_d_std;

rb_slopeint_stderr_dtrue_d =(slopeint_stderr_dtrue_d -slopeint_dtrue_d_std)/slopeint_dtrue_d_std;

rb_int_stderr_rsd_d =(int_stderr_rsd_d -int_est_rsd_d_std)/int_est_rsd_d_std;

rb_slopediff_stderr_rsd_d =(slopediff_stderr_rsd_d -slopediff_rsd_d_std)/slopediff_rsd_d_std;

rb_slopeg_stderr_rsd_d =(slopeg_stderr_rsd_d -slopeg_rsd_d_std)/slopeg_rsd_d_std;

rb_slopeint_stderr_rsd_d =(slopeint_stderr_rsd_d -slopeint_rsd_d_std)/slopeint_rsd_d_std;

rb_int_stderr_ebd_d =(int_stderr_ebd_d -int_est_ebd_d_std)/int_est_ebd_d_std;

rb_slopediff_stderr_ebd_d =(slopediff_stderr_ebd_d -slopediff_ebd_d_std)/slopediff_ebd_d_std;

rb_slopeg_stderr_ebd_d =(slopeg_stderr_ebd_d -slopeg_ebd_d_std)/slopeg_ebd_d_std;

rb_slopeint_stderr_ebd_d =(slopeint_stderr_ebd_d -slopeint_ebd_d_std)/slopeint_ebd_d_std;

rb_int_stderr_diz_d =(int_stderr_diz_d -int_est_diz_d_std)/int_est_diz_d_std;

rb_slopediff_stderr_diz_d =(slopediff_stderr_diz_d -slopediff_diz_d_std)/slopediff_diz_d_std;

rb_slopeg_stderr_diz_d =(slopeg_stderr_diz_d -slopeg_diz_d_std)/slopeg_diz_d_std;

rb_slopeint_stderr_diz_d =(slopeint_stderr_diz_d -slopeint_diz_d_std)/slopeint_diz_d_std;

rb_int_stderr_res_d =(int_stderr_res_d -int_est_res_d_std)/int_est_res_d_std;

rb_slopediff_stderr_res_d =(slopediff_stderr_res_d -slopediff_res_d_std)/slopediff_res_d_std;

rb_slopeg_stderr_res_d =(slopeg_stderr_res_d -slopeg_res_d_std)/slopeg_res_d_std;

rb_slopeint_stderr_res_d =(slopeint_stderr_res_d -slopeint_res_d_std)/slopeint_res_d_std;

rb_int_stderr_sem_d =(int_stderr_sem_d -int_est_sem_d_std)/int_est_sem_d_std;

rb_slopediff_stderr_sem_d =(slopediff_stderr_sem_d -slopediff_sem_d_std)/slopediff_sem_d_std;

rb_slopeg_stderr_sem_d =(slopeg_stderr_sem_d -slopeg_sem_d_std)/slopeg_sem_d_std;

rb_slopeint_stderr_sem_d =(slopeint_stderr_sem_d -slopeint_sem_d_std)/slopeint_sem_d_std;

if int_pvalue_dtrue_d lt .05 then int_pvalue_dtrue_count_d = 1; else int_pvalue_dtrue_count_d = 0;

if slopediff_pvalue_dtrue_d lt .05 then slopediff_pvalue_dtrue_count_d = 1; else slopediff_pvalue_dtrue_count_d = 0;

if slopeg_pvalue_dtrue_d lt .05 then slopeg_pvalue_dtrue_count_d = 1; else slopeg_pvalue_dtrue_count_d = 0;

if slopeint_pvalue_dtrue_d lt .05 then slopeint_pvalue_dtrue_count_d = 1; else slopeint_pvalue_dtrue_count_d = 0;

if int_pvalue_rsd_d lt .05 then int_pvalue_rsd_count_d = 1; else int_pvalue_rsd_count_d = 0;

if slopediff_pvalue_rsd_d lt .05 then slopediff_pvalue_rsd_count_d = 1; else slopediff_pvalue_rsd_count_d = 0;

if slopeg_pvalue_rsd_d lt .05 then slopeg_pvalue_rsd_count_d = 1; else slopeg_pvalue_rsd_count_d = 0;

if slopeint_pvalue_rsd_d lt .05 then slopeint_pvalue_rsd_count_d = 1; else slopeint_pvalue_rsd_count_d = 0;

if int_pvalue_ebd_d lt .05 then int_pvalue_ebd_count_d = 1; else int_pvalue_ebd_count_d = 0;

if slopediff_pvalue_ebd_d lt .05 then slopediff_pvalue_ebd_count_d = 1; else slopediff_pvalue_ebd_count_d = 0;

if slopeg_pvalue_ebd_d lt .05 then slopeg_pvalue_ebd_count_d = 1; else slopeg_pvalue_ebd_count_d = 0;

if slopeint_pvalue_ebd_d lt .05 then slopeint_pvalue_ebd_count_d = 1; else slopeint_pvalue_ebd_count_d = 0;

if int_pvalue_diz_d lt .05 then int_pvalue_diz_count_d = 1; else int_pvalue_diz_count_d = 0;

if slopediff_pvalue_diz_d lt .05 then slopediff_pvalue_diz_count_d = 1; else slopediff_pvalue_diz_count_d = 0;

if slopeg_pvalue_diz_d lt .05 then slopeg_pvalue_diz_count_d = 1; else slopeg_pvalue_diz_count_d = 0;

if slopeint_pvalue_diz_d lt .05 then slopeint_pvalue_diz_count_d = 1; else slopeint_pvalue_diz_count_d = 0;

if int_pvalue_res_d lt .05 then int_pvalue_res_count_d = 1; else int_pvalue_res_count_d = 0;

if slopediff_pvalue_res_d lt .05 then slopediff_pvalue_res_count_d = 1; else slopediff_pvalue_res_count_d = 0;

if slopeg_pvalue_res_d lt .05 then slopeg_pvalue_res_count_d = 1; else slopeg_pvalue_res_count_d = 0;

if slopeint_pvalue_res_d lt .05 then slopeint_pvalue_res_count_d = 1; else slopeint_pvalue_res_count_d = 0;

if int_pvalue_sem_d lt .05 then int_pvalue_sem_count_d = 1; else int_pvalue_sem_count_d = 0;

if slopediff_pvalue_sem_d lt .05 then slopediff_pvalue_sem_count_d = 1; else slopediff_pvalue_sem_count_d = 0;

if slopeg_pvalue_sem_d lt .05 then slopeg_pvalue_sem_count_d = 1; else slopeg_pvalue_sem_count_d = 0;

if slopeint_pvalue_sem_d lt .05 then slopeint_pvalue_sem_count_d = 1; else slopeint_pvalue_sem_count_d = 0;

if int_lcl_dtrue_d lt .5 and int_ucl_dtrue_d gt .5 then int_cvc_dtrue_d =1; else int_cvc_dtrue_d =0;

if slopediff_lcl_dtrue_d lt .5 and slopediff_ucl_dtrue_d gt .5 then slopediff_cvc_dtrue_d =1; else slopediff_cvc_dtrue_d =0;

if slopeg_lcl_dtrue_d lt .5 and slopeg_ucl_dtrue_d gt .5 then slopeg_cvc_dtrue_d =1; else slopeg_cvc_dtrue_d =0;

if slopeint_lcl_dtrue_d lt .5 and slopeint_ucl_dtrue_d gt .5 then slopeint_cvc_dtrue_d =1; else slopeint_cvc_dtrue_d =0;

if int_lcl_rsd_d lt .5 and int_ucl_rsd_d gt .5 then int_cvc_rsd_d =1; else int_cvc_rsd_d =0;

if slopediff_lcl_rsd_d lt .5 and slopediff_ucl_rsd_d gt .5 then slopediff_cvc_rsd_d =1; else slopediff_cvc_rsd_d =0;

if slopeg_lcl_rsd_d lt .5 and slopeg_ucl_rsd_d gt .5 then slopeg_cvc_rsd_d =1; else slopeg_cvc_rsd_d =0;

if slopeint_lcl_rsd_d lt .5 and slopeint_ucl_rsd_d gt .5 then slopeint_cvc_rsd_d =1; else slopeint_cvc_rsd_d =0;

if int_lcl_ebd_d lt .5 and int_ucl_ebd_d gt .5 then int_cvc_ebd_d =1; else int_cvc_ebd_d =0;

if slopediff_lcl_ebd_d lt .5 and slopediff_ucl_ebd_d gt .5 then slopediff_cvc_ebd_d =1; else slopediff_cvc_ebd_d =0;

if slopeg_lcl_ebd_d lt .5 and slopeg_ucl_ebd_d gt .5 then slopeg_cvc_ebd_d =1; else slopeg_cvc_ebd_d =0;

if slopeint_lcl_ebd_d lt .5 and slopeint_ucl_ebd_d gt .5 then slopeint_cvc_ebd_d =1; else slopeint_cvc_ebd_d =0;

if int_lcl_diz_d lt .5 and int_ucl_diz_d gt .5 then int_cvc_diz_d =1; else int_cvc_diz_d =0;

if slopediff_lcl_diz_d lt .5 and slopediff_ucl_diz_d gt .5 then slopediff_cvc_diz_d =1; else slopediff_cvc_diz_d =0;

if slopeg_lcl_diz_d lt .5 and slopeg_ucl_diz_d gt .5 then slopeg_cvc_diz_d =1; else slopeg_cvc_diz_d =0;

if slopeint_lcl_diz_d lt .5 and slopeint_ucl_diz_d gt .5 then slopeint_cvc_diz_d =1; else slopeint_cvc_diz_d =0;

if int_lcl_res_d lt .5 and int_ucl_res_d gt .5 then int_cvc_res_d =1; else int_cvc_res_d =0;

if slopediff_lcl_res_d lt .5 and slopediff_ucl_res_d gt .5 then slopediff_cvc_res_d =1; else slopediff_cvc_res_d =0;

if slopeg_lcl_res_d lt .5 and slopeg_ucl_res_d gt .5 then slopeg_cvc_res_d =1; else slopeg_cvc_res_d =0;

if slopeint_lcl_res_d lt .5 and slopeint_ucl_res_d gt .5 then slopeint_cvc_res_d =1; else slopeint_cvc_res_d =0;

if int_lcl_sem_d lt .5 and int_ucl_sem_d gt .5 then int_cvc_sem_d =1; else int_cvc_sem_d =0;

if slopediff_lcl_sem_d lt .5 and slopediff_ucl_sem_d gt .5 then slopediff_cvc_sem_d =1; else slopediff_cvc_sem_d =0;

if slopeg_lcl_sem_d lt .5 and slopeg_ucl_sem_d gt .5 then slopeg_cvc_sem_d =1; else slopeg_cvc_sem=0;

if slopeint_lcl_sem_d lt .5 and slopeint_ucl_sem_d gt .5 then slopeint_cvc_sem_d =1; else slopeint_cvc_sem_d =0;

rb_int_stderr_dtrue_i=(int_stderr_dtrue_i-int_est_dtrue_i_std)/int_est_dtrue_i_std;

rb_slopediff_stderr_dtrue_i=(slopediff_stderr_dtrue_i-slopediff_dtrue_i_std)/slopediff_dtrue_i_std;

rb_int_stderr_rsd_i =(int_stderr_rsd_i -int_est_rsd_i_std)/int_est_rsd_i_std;

rb_slopediff_stderr_rsd_i =(slopediff_stderr_rsd_i -slopediff_rsd_i_std)/slopediff_rsd_i_std;

rb_int_stderr_ebd_i =(int_stderr_ebd_i -int_est_ebd_i_std)/int_est_ebd_i_std;

rb_slopediff_stderr_ebd_i =(slopediff_stderr_ebd_i -slopediff_ebd_i_std)/slopediff_ebd_i_std;

rb_int_stderr_diz_i =(int_stderr_diz_i -int_est_diz_i_std)/int_est_diz_i_std;

rb_slopediff_stderr_diz_i =(slopediff_stderr_diz_i -slopediff_diz_i_std)/slopediff_diz_i_std;

rb_int_stderr_res_i =(int_stderr_res_i -int_est_res_i_std)/int_est_res_i_std;

rb_slopediff_stderr_res_i =(slopediff_stderr_res_i -slopediff_res_i_std)/slopediff_res_i_std;

rb_int_stderr_sem_i =(int_stderr_sem_i -int_est_sem_i_std)/int_est_sem_i_std;

rb_slopediff_stderr_sem_i =(slopediff_stderr_sem_i -slopediff_sem_i_std)/slopediff_sem_i_std;

if int_pvalue_dtrue_i lt .05 then int_pvalue_dtrue_count_i = 1; else int_pvalue_dtrue_count_i = 0;

if slopediff_pvalue_dtrue_i lt .05 then slopediff_pvalue_dtrue_count_i = 1; else slopediff_pvalue_dtrue_count_i = 0;

if int_pvalue_rsd_i lt .05 then int_pvalue_rsd_count_i = 1; else int_pvalue_rsd_count_i = 0;

if slopediff_pvalue_rsd_i lt .05 then slopediff_pvalue_rsd_count_i = 1; else slopediff_pvalue_rsd_count_i = 0;

if int_pvalue_ebd_i lt .05 then int_pvalue_ebd_count_i = 1; else int_pvalue_ebd_count_i = 0;

if slopediff_pvalue_ebd_i lt .05 then slopediff_pvalue_ebd_count_i = 1; else slopediff_pvalue_ebd_count_i = 0;

if int_pvalue_diz_i lt .05 then int_pvalue_diz_count_i = 1; else int_pvalue_diz_count_i = 0;

if slopediff_pvalue_diz_i lt .05 then slopediff_pvalue_diz_count_i = 1; else slopediff_pvalue_diz_count_i = 0;

if int_pvalue_res_i lt .05 then int_pvalue_res_count_i = 1; else int_pvalue_res_count_i = 0;

if slopediff_pvalue_res_i lt .05 then slopediff_pvalue_res_count_i = 1; else slopediff_pvalue_res_count_i = 0;

if int_pvalue_sem_i lt .05 then int_pvalue_sem_count_i = 1; else int_pvalue_sem_count_i = 0;

if slopediff_pvalue_sem_i lt .05 then slopediff_pvalue_sem_count_i = 1; else slopediff_pvalue_sem_count_i = 0;

if int_lcl_dtrue_i lt .5 and int_ucl_dtrue_i gt .5 then int_cvc_dtrue_i =1; else int_cvc_dtrue_i =0;

if slopediff_lcl_dtrue_i lt .5 and slopediff_ucl_dtrue_i gt .5 then slopediff_cvc_dtrue_i =1; else slopediff_cvc_dtrue_i =0;

if int_lcl_rsd_i lt .5 and int_ucl_rsd_i gt .5 then int_cvc_rsd_i =1; else int_cvc_rsd_i =0;

if slopediff_lcl_rsd_i lt .5 and slopediff_ucl_rsd_i gt .5 then slopediff_cvc_rsd_i =1; else slopediff_cvc_rsd_i =0;

if int_lcl_ebd_i lt .5 and int_ucl_ebd_i gt .5 then int_cvc_ebd_i =1; else int_cvc_ebd_i =0;

if slopediff_lcl_ebd_i lt .5 and slopediff_ucl_ebd_i gt .5 then slopediff_cvc_ebd_i =1; else slopediff_cvc_ebd_i =0;

if int_lcl_diz_i lt .5 and int_ucl_diz_i gt .5 then int_cvc_diz_i =1; else int_cvc_diz_i =0;

if slopediff_lcl_diz_i lt .5 and slopediff_ucl_diz_i gt .5 then slopediff_cvc_diz_i =1; else slopediff_cvc_diz_i =0;

if int_lcl_res_i lt .5 and int_ucl_res_i gt .5 then int_cvc_res_i =1; else int_cvc_res_i =0;

if slopediff_lcl_res_i lt .5 and slopediff_ucl_res_i gt .5 then slopediff_cvc_res_i =1; else slopediff_cvc_res_i =0;

if int_lcl_sem_i lt .5 and int_ucl_sem_i gt .5 then int_cvc_sem_i =1; else int_cvc_sem_i =0;

if slopediff_lcl_sem_i lt .5 and slopediff_ucl_sem_i gt .5 then slopediff_cvc_sem_i =1; else slopediff_cvc_sem_i =0;

run;

********************************************;

*17.TRANPOSE RSQUARE UNIVARIATE TO MULTIVAR*;

********************************************;

data fitstat2;

set fitstat;

if Label2 = 'Adj R-Sq' then delete;

if Label2 = ' ' then delete;

if Model = 'MODEL1' then iv = 'difftrue_d';

if Model = 'MODEL2' then iv = 'rsd_d';

if Model = 'MODEL3' then iv = 'ebd_d';

if Model = 'MODEL4' then iv = 'diz_d';

if Model = 'MODEL5' then iv = 'res_d';

if Model = 'MODEL6' then iv = 'sem_d';

if Model = 'MODEL7' then iv = 'difftrue_i';

if Model = 'MODEL8' then iv = 'rsd_i';

if Model = 'MODEL9' then iv = 'ebd_i';

if Model = 'MODEL10' then iv = 'diz_i';

if Model = 'MODEL11' then iv = 'res_i';

if Model = 'MODEL12' then iv = 'sem_i';

z = 1;

rsqval = cvalue2*1; /*converts rsquare value from char to numeric*/

drop Dependent Model Label1 Label2 cvalue1 nvalue1 nvalue2 ;

run;

proc transpose data = fitstat2 out = rsquare prefix = rsq_;

by iterid z;

id iv;

var rsqval;

run;

data rsquare;

set rsquare;

rsq_difftrue=rsq_difftrue*1;

drop _NAME_;

rb_rsq_rsd_d=(rsq_rsd_d-rsq_difftrue_d)/rsq_difftrue_d;

rb_rsq_ebd_d=(rsq_ebd_d-rsq_difftrue_d)/rsq_difftrue_d;

rb_rsq_diz_d=(rsq_diz_d-rsq_difftrue_d)/rsq_difftrue_d;

rb_rsq_res_d=(rsq_res_d-rsq_difftrue_d)/rsq_difftrue_d;

rb_rsq_sem_d=(rsq_sem_d-rsq_difftrue_d)/rsq_difftrue_d;

rb_rsq_rsd_i=(rsq_rsd_i-rsq_difftrue_i)/rsq_difftrue_i;

rb_rsq_ebd_i=(rsq_ebd_i-rsq_difftrue_i)/rsq_difftrue_i;

rb_rsq_diz_i=(rsq_diz_i-rsq_difftrue_i)/rsq_difftrue_i;

rb_rsq_res_i=(rsq_res_i-rsq_difftrue_i)/rsq_difftrue_i;

rb_rsq_sem_i=(rsq_sem_i-rsq_difftrue_i)/rsq_difftrue_i;

run;

data dyad.iter&sid;

merge dyad.iter&sid regparms rsquare ANOVA6;

by iterid;

run;

proc means data=dyad.iter&sid sum;

var p_difftrue_count_d

p_rsd_count_d

p_ebd_count_d

p_diz_count_d

p_res_count_d

p_sem_count_d

int_pvalue_dtrue_count_d

slopediff_pvalue_dtrue_count_d

slopeg_pvalue_dtrue_count_d

slopeint_pvalue_dtrue_count_d

int_pvalue_rsd_count_d

slopediff_pvalue_rsd_count_d

slopeg_pvalue_rsd_count_d

slopeint_pvalue_rsd_count_d

int_pvalue_ebd_count_d

slopediff_pvalue_ebd_count_d

slopeg_pvalue_ebd_count_d

slopeint_pvalue_ebd_count_d

int_pvalue_diz_count_d

slopediff_pvalue_diz_count_d

slopeg_pvalue_diz_count_d

slopeint_pvalue_diz_count_d

int_pvalue_res_count_d

slopediff_pvalue_res_count_d

slopeg_pvalue_res_count_d

slopeint_pvalue_res_count_d

int_pvalue_sem_count_d

slopediff_pvalue_sem_count_d

slopeg_pvalue_sem_count_d

slopeint_pvalue_sem_count_d

int_cvc_dtrue_d

slopediff_cvc_dtrue_d

slopeg_cvc_dtrue_d

slopeint_cvc_dtrue_d

int_cvc_rsd_d

slopediff_cvc_rsd_d

slopeg_cvc_rsd_d

slopeint_cvc_rsd_d

int_cvc_ebd_d

slopediff_cvc_ebd_d

slopeg_cvc_ebd_d

slopeint_cvc_ebd_d

int_cvc_diz_d

slopediff_cvc_diz_d

slopeg_cvc_diz_d

slopeint_cvc_diz_d

int_cvc_res_d

slopediff_cvc_res_d

slopeg_cvc_res_d

slopeint_cvc_res_d

int_cvc_sem_d

slopediff_cvc_sem_d

slopeg_cvc_sem_d

slopeint_cvc_sem_d

p_difftrue_count_i

p_rsd_count_i

p_ebd_count_i

p_diz_count_i

p_res_count_i

p_sem_count_i

int_pvalue_dtrue_count_i

slopediff_pvalue_dtrue_count_i

int_pvalue_rsd_count_i

slopediff_pvalue_rsd_count_i

int_pvalue_ebd_count_i

slopediff_pvalue_ebd_count_i

int_pvalue_diz_count_i

slopediff_pvalue_diz_count_i

int_pvalue_res_count_i

slopediff_pvalue_res_count_i

int_pvalue_sem_count_i

slopediff_pvalue_sem_count_i

int_cvc_dtrue_i

slopediff_cvc_dtrue_i

int_cvc_rsd_i

slopediff_cvc_rsd_i

int_cvc_ebd_i

slopediff_cvc_ebd_i

int_cvc_diz_i

slopediff_cvc_diz_i

int_cvc_res_i

slopediff_cvc_res_i

int_cvc_sem_i

slopediff_cvc_sem_i

;

output out=power(drop= _FREQ_ _TYPE_) sum=sum_p_difftrue_count_d

sum_p_rsd_count_d

sum_p_ebd_count_d

sum_p_diz_count_d

sum_p_res_count_d

sum_p_sem_count_d

sum_ int_pvalue_dtrue_count_d

sum_slpdiff_pvalue_dtrue_count_d

sum_slopeg_pvalue_dtrue_count_d

sum_slpint_pvalue_dtrue_count_d

sum_int_pvalue_rsd_count_d

sum_slopediff_pvalue_rsd_count_d

sum_slopeg_pvalue_rsd_count_d

sum_slopeint_pvalue_rsd_count_d

sum_int_pvalue_ebd_count_d

sum_slopediff_pvalue_ebd_count_d

sum_slopeg_pvalue_ebd_count_d

sum_slopeint_pvalue_ebd_count_d

sum_int_pvalue_diz_count_d

sum_slopediff_pvalue_diz_count_d

sum_slopeg_pvalue_diz_count_d

sum_slopeint_pvalue_diz_count_d

sum_int_pvalue_res_count_d

sum_slopediff_pvalue_res_count_d

sum_slopeg_pvalue_res_count_d

sum_slopeint_pvalue_res_count_d

sum_int_pvalue_sem_count_d

sum_slopediff_pvalue_sem_count_d

sum_slopeg_pvalue_sem_count_d

sum_slopeint_pvalue_sem_count_d

sum_int_cvc_dtrue_d

sum_slopediff_cvc_dtrue_d

sum_slopeg_cvc_dtrue_d

sum_slopeint_cvc_dtrue_d

sum_int_cvc_rsd_d

sum_slopediff_cvc_rsd_d

sum_slopeg_cvc_rsd_d

sum_slopeint_cvc_rsd_d

sum_int_cvc_ebd_d

sum_slopediff_cvc_ebd_d

sum_slopeg_cvc_ebd_d

sum_slopeint_cvc_ebd_d

sum_int_cvc_diz_d

sum_slopediff_cvc_diz_d

sum_slopeg_cvc_diz_d

sum_slopeint_cvc_diz_d

sum_int_cvc_res_d

sum_slopediff_cvc_res_d

sum_slopeg_cvc_res_d

sum_slopeint_cvc_res_d

sum_int_cvc_sem_d

sum_slopediff_cvc_sem_d

sum_slopeg_cvc_sem_d

sum_slopeint_cvc_sem_d

sum_p_difftrue_count_i

sum_p_rsd_count_i

sum_p_ebd_count_i

sum_p_diz_count_i

sum_p_res_count_i

sum_p_sem_count_i

sum_int_pvalue_dtrue_count_i

sum_slpdiff_pvalue_dtrue_count_i

sum_int_pvalue_rsd_count_i

sum_slopediff_pvalue_rsd_count_i

sum_int_pvalue_ebd_count_i

sum_slopediff_pvalue_ebd_count_i

sum_int_pvalue_diz_count_i

sum_slopediff_pvalue_diz_count_i

sum_int_pvalue_res_count_i

sum_slopediff_pvalue_res_count_i

sum_int_pvalue_sem_count_i

sum_slopediff_pvalue_sem_count_i

sum_int_cvc_dtrue_i

sum_slopediff_cvc_dtrue_i

sum_int_cvc_rsd_i

sum_slopediff_cvc_rsd_i

sum_int_cvc_ebd_i

sum_slopediff_cvc_ebd_i

sum_int_cvc_diz_i

sum_slopediff_cvc_diz_i

sum_int_cvc_res_i

sum_slopediff_cvc_res_i

sum_int_cvc_sem_i

sum_slopediff_cvc_sem_i

;

run;

proc means data=dyad.outb&sid noprint mean std;

output out=dyad.sum&sid mean=iterID_mean

grandslope_mean

dyadID_mean

true1_mean

true2_mean

simid_mean

difftrue_mean

difftrueabs_mean

dyadmean_mean

group_mean

raw1_mean

raw2_mean

rsd_mean

rawgroup_mean

rsdabs_mean

rbrsd_mean

absbiasraw1_mean

absbiasraw2_mean

rawdyadmean_mean

meanraw1_mean

meanraw2_mean

stdraw1_mean

stdraw2_mean

varraw1_mean

varraw2_mean

zraw1_mean

zraw2_mean

diz_mean

meantrue1_mean

meantrue2_mean

stdtrue1_mean

stdtrue2_mean

vartrue1_mean

vartrue2_mean

ztrue1_mean

ztrue2_mean

diztrue_mean

rbdiz_mean

dizabs_mean

praw1_mean

res_mean

ptrue1_mean

restrue_mean

rbres_mean

resabs_mean

dyadint_mean

dyadslope_mean

ebd_mean

ebdabs_mean

rbebd_mean

sem_mean

semabs_mean

rbsem_mean

outcome_mean

rsq_theor_mean

difftrueabsg_mean

rsdabsg_mean

dizabsg_mean

resabsg_mean

ebdabsg_mean

semabsg_mean

std=iterID_std

grandslope_std

dyadID_std

true1_std

true2_std

simid_std

difftrue_std

difftrueabs_std

dyadmean_std

group_std

raw1_std

raw2_std

rsd_std

rawgroup_std

rsdabs_std

rbrsd_std

absbiasraw1_std

absbiasraw2_std

rawdyadmean_std

meanraw1_std

meanraw2_std

stdraw1_std

stdraw2_std

varraw1_std

varraw2_std

zraw1_std

zraw2_std

diz_std

meantrue1_std

meantrue2_std

stdtrue1_std

stdtrue2_std

vartrue1_std

vartrue2_std

ztrue1_std

ztrue2_std

diztrue_std

rbdiz_std

dizabs_std

praw1_std

res_std

ptrue1_std

restrue_std

rbres_std

resabs_std

dyadint_std

dyadslope_std

ebd_std

ebdabs_std

rbebd_std

sem_std

semabs_std

rbsem_std

outcome_std

rsq_theor_std

difftrueabsg_std

rsdabsg_std

dizabsg_std

resabsg_std

ebdabsg_std

semabsg_std;

run;

data dyad.sum&sid;

merge dyad.sum&sid power;

power_difftrue_d=sum_p_difftrue_count_d/&NumSamples;

power_rsd_d=sum_p_rsd_count_d/&NumSamples;

power_ebd_d=sum_p_ebd_count_d/&NumSamples;

power_diz_d=sum_p_diz_count_d/&NumSamples;

power_res_d=sum_p_res_count_d/&NumSamples;

power_sem_d=sum_p_sem_count_d/&NumSamples;

power_int_pvalue_dtrue_d=sum_int_pvalue_dtrue_count_d/&NumSamples;

power_slpdiff_pvalue_dtrue_d=sum_slpdiff_pvalue_dtrue_count_d/&NumSamples;

power_slopeg_pvalue_dtrue_d=sum_slopeg_pvalue_dtrue_count_d/&NumSamples;

power_slopeint_pvalue_dtrue_d=sum_slpint_pvalue_dtrue_count_d/&NumSamples;

power_int_pvalue_rsd_d=sum_int_pvalue_rsd_count_d/&NumSamples;

power_slopediff_pvalue_rsd_d=sum_slopediff_pvalue_rsd_count_d/&NumSamples;

power_slopeg_pvalue_rsd_d=sum_slopeg_pvalue_rsd_count_d/&NumSamples;

power_slopeint_pvalue_rsd_d=sum_slopeint_pvalue_rsd_count_d/&NumSamples;

power_int_pvalue_ebd_d=sum_int_pvalue_ebd_count_d/&NumSamples;

power_slopediff_pvalue_ebd_d=sum_slopediff_pvalue_ebd_count_d/&NumSamples;

power_slopeg_pvalue_ebd_d=sum_slopeg_pvalue_ebd_count_d/&NumSamples;

power_slopeint_pvalue_ebd_d=sum_slopeint_pvalue_ebd_count_d/&NumSamples;

power_int_pvalue_diz_d=sum_int_pvalue_diz_count_d/&NumSamples;

power_slopediff_pvalue_diz_d=sum_slopediff_pvalue_diz_count_d/&NumSamples;

power_slopeg_pvalue_diz_d=sum_slopeg_pvalue_diz_count_d/&NumSamples;

power_slopeint_pvalue_diz_d=sum_slopeint_pvalue_diz_count_d/&NumSamples;

power_int_pvalue_res_d=sum_int_pvalue_res_count_d/&NumSamples;

power_slopediff_pvalue_res_d=sum_slopediff_pvalue_res_count_d/&NumSamples;

power_slopeg_pvalue_res_d=sum_slopeg_pvalue_res_count_d/&NumSamples;

power_slopeint_pvalue_res_d=sum_slopeint_pvalue_res_count_d/&NumSamples;

power_int_pvalue_sem_d=sum_int_pvalue_sem_count_d/&NumSamples;

power_slopediff_pvalue_sem_d=sum_slopediff_pvalue_sem_count_d/&NumSamples;

power_slopeg_pvalue_sem_d=sum_slopeg_pvalue_sem_count_d/&NumSamples;

power_slopeint_pvalue_sem_d=sum_slopeint_pvalue_sem_count_d/&NumSamples;

int_dtrue_covrate_d=sum_int_cvc_dtrue_d/&NumSamples;

slopediff_dtrue_covrate_d=sum_slopediff_cvc_dtrue_d/&NumSamples;

slopeg_dtrue_covrate_d=sum_slopeg_cvc_dtrue_d/&NumSamples;

slopeint_dtrue_covrate_d=sum_slopeint_cvc_dtrue_d/&NumSamples;

int_rsd_covrate_d=sum_int_cvc_rsd_d/&NumSamples;

slopediff_rsd_covrate_d=sum_slopediff_cvc_rsd_d/&NumSamples;

slopeg_rsd_covrate_d=sum_slopeg_cvc_rsd_d/&NumSamples;

slopeint_rsd_covrate_d=sum_slopeint_cvc_rsd_d/&NumSamples;

int_ebd_covrate_d=sum_int_cvc_ebd_d/&NumSamples;

slopediff_ebd_covrate_d=sum_slopediff_cvc_ebd_d/&NumSamples;

slopeg_ebd_covrate_d=sum_slopeg_cvc_ebd_d/&NumSamples;

slopeint_ebd_covrate_d=sum_slopeint_cvc_ebd_d/&NumSamples;

int_diz_covrate_d=sum_int_cvc_diz_d/&NumSamples;

slopediff_diz_covrate_d=sum_slopediff_cvc_diz_d/&NumSamples;

slopeg_diz_covrate_d=sum_slopeg_cvc_diz_d/&NumSamples;

slopeint_diz_covrate_d=sum_slopeint_cvc_diz_d/&NumSamples;

int_res_covrate_d=sum_int_cvc_res_d/&NumSamples;

slopediff_res_covrate_d=sum_slopediff_cvc_res_d/&NumSamples;

slopeg_res_covrate_d=sum_slopeg_cvc_res_d/&NumSamples;

slopeint_res_covrate_d=sum_slopeint_cvc_res_d/&NumSamples;

int_sem_covrate_d=sum_int_cvc_sem_d/&NumSamples;

slopediff_sem_covrate_d=sum_slopediff_cvc_sem_d/&NumSamples;

slopeg_sem_covrate_d=sum_slopeg_cvc_sem_d/&NumSamples;

slopeint_sem_covrate_d=sum_slopeint_cvc_sem_d/&NumSamples;

power_difftrue_d=sum_p_difftrue_count_i/&NumSamples;

power_rsd_i=sum_p_rsd_count_i/&NumSamples;

power_ebd_i=sum_p_ebd_count_i/&NumSamples;

power_diz_i=sum_p_diz_count_i/&NumSamples;

power_res_i=sum_p_res_count_i/&NumSamples;

power_sem_i=sum_p_sem_count_i/&NumSamples;

power_int_pvalue_dtrue_i=sum_int_pvalue_dtrue_count_i/&NumSamples;

power_slpdiff_pvalue_dtrue_i=sum_slpdiff_pvalue_dtrue_count_i/&NumSamples;

power_int_pvalue_rsd_i=sum_int_pvalue_rsd_count_i/&NumSamples;

power_slopediff_pvalue_rsd_i=sum_slopediff_pvalue_rsd_count_i/&NumSamples;

power_int_pvalue_ebd_i=sum_int_pvalue_ebd_count_i/&NumSamples;

power_slopediff_pvalue_ebd_i=sum_slopediff_pvalue_ebd_count_i/&NumSamples;

power_int_pvalue_diz_i=sum_int_pvalue_diz_count_i/&NumSamples;

power_slopediff_pvalue_diz_i=sum_slopediff_pvalue_diz_count_i/&NumSamples;

power_int_pvalue_res_i=sum_int_pvalue_res_count_i/&NumSamples;

power_slopediff_pvalue_res_i=sum_slopediff_pvalue_res_count_i/&NumSamples;

power_int_pvalue_sem_i=sum_int_pvalue_sem_count_i/&NumSamples;

power_slopediff_pvalue_sem_i=sum_slopediff_pvalue_sem_count_i/&NumSamples;

int_dtrue_covrate_i=sum_int_cvc_dtrue_i/&NumSamples;

slopediff_dtrue_covrate_i=sum_slopediff_cvc_dtrue_i/&NumSamples;

int_rsd_covrate_i=sum_int_cvc_rsd_i/&NumSamples;

slopediff_rsd_covrate_i=sum_slopediff_cvc_rsd_i/&NumSamples;

int_ebd_covrate_i=sum_int_cvc_ebd_i/&NumSamples;

slopediff_ebd_covrate_i=sum_slopediff_cvc_ebd_i/&NumSamples;

int_diz_covrate_i=sum_int_cvc_diz_i/&NumSamples;

slopediff_diz_covrate_i=sum_slopediff_cvc_diz_i/&NumSamples;

int_res_covrate_i=sum_int_cvc_res_i/&NumSamples;

slopediff_res_covrate_i=sum_slopediff_cvc_res_i/&NumSamples;

int_sem_covrate_i=sum_int_cvc_sem_i/&NumSamples;

slopediff_sem_covrate_i=sum_slopediff_cvc_sem_i/&NumSamples;

simid=&simid;

/*N = &N; */

/*NumSamples = &NumSamples;*/

/*meandiff=&meandiff; */

/*var1=&var1;*/

/*var2=&var2;*/

/*corr=&corr;*/

/*meana=&meana;*/

/*meanb=&meanb;*/

/*cov=&cov;*/

/*d=&d; */

run;

%end;

%let id=sysfunc(close(&id));

%mend;

%doit;

**Appendix 1-D Explanation of data generation model**:

The table below shows the components manipulated to yield the desired characteristics of each simulation condition. Each component is explained below the table. The calculations are illustrated using desired values from simulation condition #25, which are:

- N of dyads = 50
- ICC = 0.3
- Effect Size = 0.2
- Effect Size Variance = 0.5
- Reliability = 0.7

| Dyad ID | Dyad Mean | Within Dyad Error | Individual True Score | Measurement Error | Individual Raw Score | Effect Size | Individual Raw Score with Effect Size Added |
| --- | --- | --- | --- | --- | --- | --- | --- |
| 1 | a | x1 | = a + x1 | e1 | a + x1 + e1 | es1 | a + x1 + e1 + es1 |
| 1 | a | x2 | = a + x2 | e2 | a + x2 + e2 |  | a + x2 + e2 |
| 2 | b | x3 | = b + x3 | e3 | b + x3 + e3 | es2 | b + x3 + e3 + es2 |
| 2 | b | x4 | = b + x4 | e4 | b + x4 + e4 |  | b + x4 + e4 |
| 3 | c | x5 | = c + x5 | e5 | c + x5 + e5 | es3 | c + x5 + e5 + es3 |
| 3 | c | x6 | = c + x6 | e6 | c + x6 + e6 |  | c + x6 + e6 |

*Dyad Means* were normally distributed with a mean of 0 and variance (bvar) set to the appropriate value to yield the desired ICC. *Within Dyad Error* values were normally distributed with a mean of 0 and variance (wvar) set to the appropriate value to yield the desired ICC.

The variances for *Dyad Means* and *Within Dyad Error* (between variance "bvar" and within variance "wvar") were set according to the desired ICC. If ICC was equal to between variance divided by total variance, and total variance was fixed to 1, between and within variance could be solved for.

Example: If we want to fix ICC to 0.3, we can solve for bvar (ICC of 0.3 = bvar / total var --> bvar = 0.3 / 1 --> bvar = 0.3

After solving for bvar, we can solve for wvar. Total variance = bvar + wvar --> 1 = 0.3 + wvar --> wvar = 0.7

Once the *Dyad Means* and *Within Dyad Errors* were generated, they were summed to yield the Individual True Score. The variance of *Dyad Means* is τ_00_ in Equation 4c, the variance of *Within Dyad Errors* is δ^2^ in Equation 3b.

To get *Individual Raw Scores, Measurement Error* (aka error variance) was generated with a mean of 0 and variance set to the value needed to yield the desired reliability. If reliability was true variance divided by the sum of true and error variance, and true variance was fixed to 1, error variance could be solved for.

Example: If we want to fix reliability to 0.7 per our simulation condition, we can solve for error variance (reliability of 0.7 = 1 / (1 + error variance --> error variance = 0.4285). Therefore, the error values were generated with a mean of 0 and variance of 0.4285.

Finally, the *Individual True Score* was added to *Measurement Error* to get the *Individual Raw Score*. At this point in the data generation process, we have generated all the components necessary to yield raw scores with a mean of 0. Effect size was added in the subsequent step.

Effect size was randomly generated with both mean and variance set according to the simulation condition. For example, if Effect Size was 0.2 and Effect Size Variance was 0.5, the distribution of Effect Sizes had a mean of 0.2 and a variance of 0.5.

The effect size values were then randomly added to one individual in each dyad, resulting in pairs of scores with a mean discrepancy of 0.2 with variance of 0.5.
